# Virtual and Biophysical Screening Targeting the γ-Tubulin Complex – A New Target for the Inhibition of Microtubule Nucleation

**DOI:** 10.1371/journal.pone.0063908

**Published:** 2013-05-15

**Authors:** Olivier Cala, Marie-Hélène Remy, Valérie Guillet, Andreas Merdes, Lionel Mourey, Alain Milon, Georges Czaplicki

**Affiliations:** 1 Institut de Pharmacologie et de Biologie Structurale, UMR 5089 CNRS – Université de Toulouse, UPS, Toulouse, France; 2 Centre de Biologie du Développement, CNRS, Toulouse, France; 3 Faculté des Sciences et de l’Ingénierie, Université de Toulouse, UPS, Toulouse, France; UMR-S665, INSERM, Université Paris Diderot, INTS, France

## Abstract

Microtubules are the main constituents of mitotic spindles. They are nucleated in large amounts during spindle assembly, from multiprotein complexes containing γ-tubulin and associated γ-tubulin complex proteins (GCPs). With the aim of developing anti-cancer drugs targeting these nucleating complexes, we analyzed the interface between GCP4 and γ-tubulin proteins usually located in a multiprotein complex named γ-TuRC (γ-Tubulin Ring Complex). 10 ns molecular dynamics simulations were performed on the heterodimers to obtain a stable complex *in silico* and to analyze the residues involved in persistent protein-protein contacts, responsible for the stability of the complex. We demonstrated *in silico* the existence of a binding pocket at the interface between the two proteins upon complex formation. By combining virtual screening using a fragment-based approach and biophysical screening, we found several small molecules that bind specifically to this pocket. Sub-millimolar fragments have been experimentally characterized on recombinant proteins using differential scanning fluorimetry (DSF) for validation of these compounds as inhibitors. These results open a new avenue for drug development against microtubule-nucleating γ-tubulin complexes.

## Introduction

Physiological or pathological processes are mainly controlled by protein–protein interactions (PPIs) [1], [2], which constitute a promising but difficult pharmacological target in many diseases. Analysis of the interface between proteins might be of crucial interest in finding new binding sites and thus potential new drugs acting by complex destabilization. It has been shown that several residues of each protein are usually involved in the interaction. In the drug discovery process, two key issues have to be resolved: to determine a biological target and to characterize a therapeutic component, which binds to a specific binding site, capable of modulating the target activity. In the past decade, the fragment based lead discovery (FBLD) has emerged to help finding new drugs [3]–[5]. The first step in FBLD is to develop fragment libraries. They should be small and should respect physical properties according to the rules of five or of three [6], [7]. These compounds can interact with the target with a weak affinity (high µM to mM range). The final step could be the combination of the best small elements in order to build a single molecular entity with affinity higher than that of the individual components.

This strategy offers possibilities of cancer chemotherapy, affecting microtubule dynamics and thus causing errors in the assembly of mitotic spindles, leading to cell cycle arrest [8]. Spindle microtubules in all eukaryotes are nucleated from multiprotein complexes. It was clearly shown that complexes of γ-tubulin are involved in microtubule nucleation [9]–[12]. An evolutionarily conserved heterotetramer forms the scaffold of this complex, composed of two copies of γ-tubulin and one of each of GCP2 and GCP3, called γ-tubulin small complex (γ-TuSC) [13]. In most eukaryotic cells, multiple γ-TuSCs associate with GCP4, GCP5 and GCP6 into a large complex of 2 MDa called γ-tubulin ring complex (γ-TuRC) [14], [15]. Refined cryo-electron microscopy has revealed that the γ-TuRCs form short helical assemblies rather than ‘rings’ [16], [17]. It is thought that the γ-TuRCs act as templates that nucleate the 13 tubulin protofilaments from dimers of α- and β-tubulin. These protofilaments establish lateral contacts to form the hollow microtubules [16].

Proteins of the GCP family show limited sequence homology [13], [15]. Structural analysis of GCP4, combined with modeling, indicated that the core structure of all GCP family members is similar and that the GCP4 structure, as the prototype, can replace GCP2 or GCP3 in an EM density model of the γ-TuSC [18]. In addition, biochemical results show that GCP4 interacts with γ-tubulin via its C-terminal domain, as previously shown for GCP2 and GCP3 [18], [19].

Several drugs such as paclitaxel derivatives and vinca alkaloids are now routinely used in chemotherapy of cancer, affecting dynamics of microtubules and thus provoking errors in mitotic spindle assembly. The targets of these inhibitors are α- and β-tubulin [8]. So far no chemotherapeutic agents have been developed against γ-tubulin or against any of its associated γ-tubulin complex proteins except some drug-like compounds recently shown to interact with γ-tubulin [20]. Nevertheless, removal of γ-tubulin or of other γ-TuRC components from the cell induces changes in microtubule dynamics and spindle defects that resemble phenotypes obtained with microtubule drugs [21]–[23]. It will therefore be of major interest to investigate the assembly of γ-TuRCs, since these are crucial for microtubule assembly and may represent potential pharmacological targets: Whitehurst et al. [24] have described proteins of the γ-TuRC as putative targets whose depletion by siRNA sensitizes a lung cancer cell line at 1000-fold reduced doses of paclitaxel. Moreover, it has been shown that small amounts of siRNA against the γ-TuRC component NEDD1 potentiate the anti-mitotic activity of low doses of a Plk1 inhibitor [23].

In this study, we targeted the discovery of ligands that bind specifically at the interface of γ-tubulin and GCP4 proteins. This was possible thanks to the recently solved 3D structures of each of the proteins and the modeling of the γ-tubulin complex [18], [25], [26]. Biochemical interaction study of various GCP4 mutants selected from structure analysis highlighted residues involved in the interaction with γ-tubulin. Molecular dynamics simulations allowed obtaining a stable conformation of the complex in agreement with previous results of Kollman et al. [17]. We discovered a promising binding pocket at the interface between the two proteins and existing only after complex formation. This binding pocket has been used to find a potential inhibitor of this complex using FBLD. Sub-millimolar interaction has been demonstrated experimentally using differential scanning fluorimetry (DSF). These results are promising in search for new drugs that inhibit the interaction between GCP proteins and γ-tubulin and thus destabilize existing γ-TuRCs or prevent the assembly of its components. Since intact γ-TuRCs are essential for microtubule nucleation and mitosis, such drugs are expected to interfere with those mechanisms thus provoking mitotic spindle defects and arrest of the cell cycle.

## Materials and Methods

### Model Building of the GCP4-γ-tubulin Complex

The GCP4 crystal structure (PDB code 3RIP) was fitted into the yeast 8 Å cryo-electron microscopy (EM) reconstruction of the *Saccharomyces cerevisiae* γ–tubulin small complex (γ-TuSC) [18], along with the crystal structure of human γ-tubulin (PDB code 3CB2) already available. A pseudo-atomic model of the γ-TuSC was generated with GCP4 crystal structure as stands-in for yeast GCP2 and GCP3 (*S. cerevisiae* Spc97 and Spc98, respectively).

GCP4 fits remarkably well into the γ-TuSC cryo-EM structure. Some manual adjustments were necessary in the bend angle between the third and fourth helical bundles with relative rearrangements of N- and C-terminal domains. The obtained model revealed interaction surfaces between the complex components. Since some loops were missing in the original GCP4 crystal structure, they were added using the SuperLooper Web server [27]. Since the insertion of imported loop fragments may have induced a certain strain, a simulation of molecular dynamics has been used to allow the structure to relax and to adopt a preferential conformation in the complex. The details of the procedure are described in the next section.

### Molecular Dynamics

The starting point for the MD simulations was the homology model of a GCP4-γ-tubulin tetramer [16]–[18]. Our objective was to verify the stability of the complex during a long MD run and to extract details on persistent intermolecular contacts from the MD trajectories.

The simulations were performed with the Amber9 software [28], using the all-atom ff03 force field [29] and the TIP3P water model [30]. A periodic system was created, containing the tetramer, 44 Na+ ions and 96432 water molecules, filling a box whose sizes were 138 × 143 × 176 Å in the X, Y and Z directions, respectively. The system minimization and the molecular dynamics simulations were performed using the parallel version of the PMEMD program. At first, the energy of the system was minimized by 200 cycles of the steepest descent (SD) algorithm, with the solute held fixed, by constraining its Cartesian coordinates using a harmonic potential with the force constant *k* equal to 500 kcal/mol/Å^2^. In the second step, the energy was minimized by 200 cycles of SD and 1000 cycles of the conjugate gradients (CG) algorithm, with weakly restrained solute (*k* = 10 kcal/mol/Å^2^). Next, a short 20 ps MD run was performed on weakly restrained solute (*k* = 50 kcal/mol/Å^2^) with temperature varying linearly from 0 to 300 K. The temperature control was achieved using the Langevin dynamics with the collision frequency parameter γ equal to 1.0 ps^−1^. The integration step used in this run was 1 fs. Throughout the calculations a cutoff of 12 Å was used for electrostatic interactions. The MD simulation continued for 180 ps at constant pressure of 1 bar and at 300 K with no restraints, with the integration step of 2 fs. The Langevin dynamics was used to control the temperature, with γ = 1.0 ps^−1^, while the pressure was controlled by isotropic scaling with the pressure relaxation time τ_p_ = 2 ps. Bonds involving hydrogen were constrained with the SHAKE algorithm. Finally, an MD simulation of 10 ns at constant pressure of 1 bar and at 300 K was launched, with atomic coordinates saved every 5 ps. The calculation was performed in parallel on two PowerEdge R410 servers, 16 cores in total, which worked at the speed of ca. 0.25 ns/day. Attempts to launch the calculation on more cores led to a slower computation, mainly due to the bottlenecks in the Gigabit Ethernet network.

### Virtual Screening

Docking studies were performed using AutoDockTools [31] v1.5.4 and AutoDockVina [32]. The coordinate pdbqt file for γ-TuSC was prepared from the last frame of the molecular dynamics trajectory, using AutoDockTools v1.5.4. We used the last frame following a clustering analysis (*kclust* from the MMTSB toolset) of the last 2 ns of the trajectory, which showed that the frames formed only one single cluster with the dispersion of 1.5 Å with respect to the centroid. Since AutoDock Vina uses a united-atom scoring function, only polar hydrogens were retained, and partial charges were calculated for the protein according to the Kollman method. A grid box was built around the complex with the sizes of 120 Å, 82 Å and 70 Å in the x, y, and z dimensions, respectively. A spacing of 1 Å between the grid points was used, placing the interface of the chain B of GCP4 and the chain D of γ-tubulin to be at the center of the cube, i.e. x, y, and z offsets of −6.538 Å, 20.792 Å, and 28.966 Å, respectively. For the binding pocket, a grid box was built with x, y, and z sizes of 40 Å, 52 Å and 40 Å, respectively. A spacing of 1 Å between the grid points was used, placing the binding pocket to be the center of the cube, with x, y, and z centers at 19.821 Å, 28.209 Å, and 8.286 Å, respectively.

Fragments were provided by the Enamine chemical libraries. 50,000 molecules were sorted according to the rules of five of Lipinski to get 500 fragments in SD files. These files were translated to 3-dimensional coordinates in PDB format using Open Babel 2.2.3 [33]. Individual PDB files for each ligand were prepared for docking using rigid and flexible side chains for amino acids involved in the pocket. Partial charges were computed according to the Gasteiger's method, non-polar hydrogens were merged and rotatable bonds were set using the prepare_ligand4.py script from MGLTools 1.5.4 [31].

A total of eight CPUs were used to perform the docking. Docking was carried out with the exhaustiveness value of 20 and the maximum output of 20 structures. For all other parameters we have used default values as defined by AutoDock Vina. The resulting conformations have been analyzed to find the most preferred ligands (clusters with a maximum number of conformations and minimum energy) in each case.

The resulting docking poses were visualized and overlaid with PyMol v0.99rc6.

The three-dimensional structure of GCP4 in one mutated form was generated by homology modeling, thus simulating the structural consequences of the S623R mutation on GCP4. For the evaluation of the results, the Deep-View analysis tool was used. (http://www.expasy.org/spdbv/, Swiss Institute of Bioinformatics, Geneva, Switzerland) [34]. Energy minimization was performed with the partial implementation of the GROMOS96 force-field using the steepest descent and conjugate gradient technique to correct the stereochemistry of the model.

### Protein Expression and Purification

Expression of GCP4 and GCP4 S623R mutant was performed as described by Studier et al. [35]. BL21 (DE3) carrying a target plasmid was grown at 30°C with shaking at 190 rpm in LB medium supplemented with kanamycin at 100 µg/ml, and chloramphenicol at 25 µg/ml. The overnight cultures (10 mL) were collected by centrifugation when the optical density at 600 nm reached 0.8 and washed once with M9 medium without a carbon source. The washed bacteria were transferred to 1 L of M9 medium supplemented with 40 mL of carbon source 25X, 10 mL trace metals 100X, 1 mL vitamins mixed 1000X and kanamycin at 100 µg/ml final and grown at 20°C with agitation at 190 rpm for 3 days. Growth was monitored by measuring the optical density at 600 nm.

Purification of native and mutant proteins was carried out as described in Guillet et al. [18] at 4°C. Cells were resuspended in lysis buffer (50 mM sodium phosphate, pH 8.0, 300 mM NaCl, 10 mM imidazole, 5% glycerol) with a protease inhibitor cocktail, 2.5 mM tris-(2-carboxyethyl)phosphine (TCEP) and benzonase (5 U/mL culture) for 1 h and were lysed by sonication by applying five 30-s pulses. Cell debris was pelleted by centrifugation at 20,000 *g* for 40 min. The supernatant was diluted 1/5 in phosphate buffer (50 mM sodium phosphate, pH 8.0, 150 mM NaCl, 5% glycerol and 0.5 mM TCEP) supplemented with 10 mM imidazole and was then loaded onto a 5-ml HisTrap FF column (GE Healthcare). The column was washed first with phosphate buffer supplemented with 10 mM imidazole until the absorbance at 280 nm reached zero, then with 50 mM imidazole in phosphate buffer to nonspecifically elute proteins bound to the column. Recombinant proteins were eluted at 150 mM imidazole in phosphate buffer. The recombinant proteins were further purified by size-exclusion chromatography using a Superdex 200 16/60 column (GE Healthcare) equilibrated in gel filtration buffer (50 mM Tris, pH 8.2, 300 mM NaCl and 2 mM DTT).

The human *TUBG1* gene was inserted into a pET15b vector between the *XhoI* and *HindIII* restriction sites. The identity of the mutation and the correctness of gene insertion (location and orientation) were verified by DNA sequencing. The recombinant protein was expressed in *Escherichia coli BL21 (DE3)* host cells in LB medium supplemented with 100 µg/ml ampicillin. The cultures were grown at 25°C until OD600 reached a value of 0.8 and then induced with 1.0 mM IPTG for 18 h. Subsequently, the cells were harvested by centrifugation (7,000 rpm for 20 min in JS 7.5 rotor). The γ-tubulin protein was then isolated and purified from the inclusion bodies via refolding by dilution with immobilized metal ion affinity chromatography (IMAC) using a Ni-NTA column according to the protocol published by Friesen et al. [20]. The recombinant protein was further purified by size-exclusion chromatography using a Superdex 200 16/60 column (GE Healthcare), equilibrated in gel filtration buffer (50 mM Tris, pH 8.2, 300 mM NaCl and 2 mM DTT).

### Differential Scanning Fluorimetry (DSF)

The DSF compound screen generally followed the protocol published by Niesen et al. [36]. Samples were loaded into a white/clear 96-well PCR plate (Bio-Rad) with each well containing a final volume of 20 µl. The concentration of protein in each well was 5 µM (0.4 mg/ml) for GCP4 or GCP4 S623R, 5 µM (0.27 mg/ml) for γ-tubulin in 50 mM Tris, pH 8.2, 300 mM NaCl, and 5X SYPRO Orange (Invitrogen). The purity of selected compounds from Enamine has been verified by NMR. They were used at a concentration from 1 µM to 1 mM. The PCR plates were sealed with optical quality sealing tape (Bio-Rad).

DSF experiments were carried out using a CFX96 real-time PCR system (Bio-Rad) set to use the 480/500 excitation and 560/580 emission filters. The samples were heated from 20 to 89.9°C at the rate of 6°C/min. A single fluorescence measurement was taken every 0.3°C. Melting temperatures were determined by performing a curve fit to the Boltzmann equation. The degree of thermal shift was calculated by comparing the melting temperature of the protein in each condition.

## Results

### Molecular Dynamics

**Figure 1:**
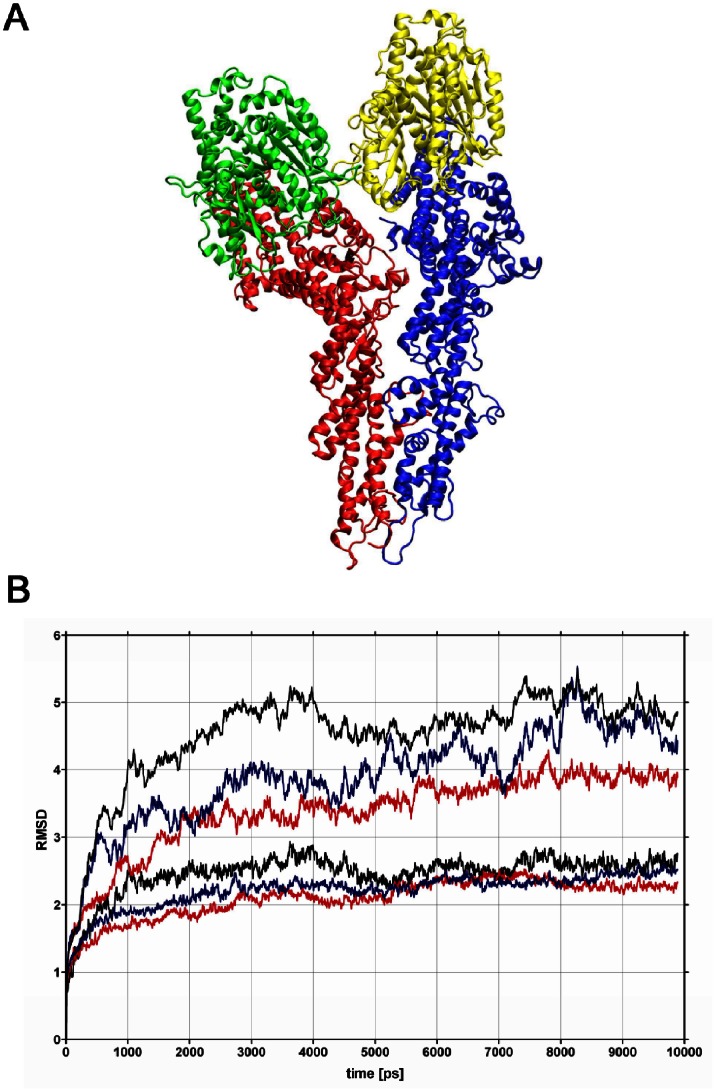
The (GCP4-γ-tubulin)_2_ tetramer. (a) The starting structure of the tetramer. The four molecules are represented by different colors: dimer 1 with red (GCP4) and green (γ-tubulin), dimer 2 with blue (GCP4) and yellow (γ-tubulin). (b) RMSD of the atomic coordinates in the (GCP4-γ-tubulin)_2_ tetramer as a function of time. Color coding: black for all atoms (upper trace) and for atoms from the intermolecular interface (lower trace), blue for dimer AC (upper trace GCP4, lower trace γ-tubulin), red for dimer BD (upper trace GCP4, lower trace γ- tubulin).

The starting point for the molecular dynamics (MD) simulations was the homology model of the GCP4-γ-tubulin tetramer [16]–[18]. Our objective was to verify the stability of the complex during a long MD run and to extract details on persistent intermolecular contacts from the MD trajectory. On top of standard equilibrium parameters such as energy, volume, density and pressure of the system (data not shown), we followed the evolution of the root mean square deviation (RMSD) of the atomic coordinates of the whole system, as well as of its components. The system evolved in time from the initial, unoptimized model of the tetramer to the equilibrated and stable complex. Figure 1a shows the initial structure of the model, used as input in the MD simulation. Figure 1b shows the variation of RMSD of the system and its components as a function of time, calculated from a superposition of atomic coordinates for each frame of the trajectory with respect to the first of the saved frames (the first frame represents the complex before minimization and equilibration). The results have been corrected for the overall rotation and translation of the whole complex. It can be seen that the system needed ca. 3 ns to equilibrate. The remaining 7 ns were used for trajectory analysis.

**Figure 2:**
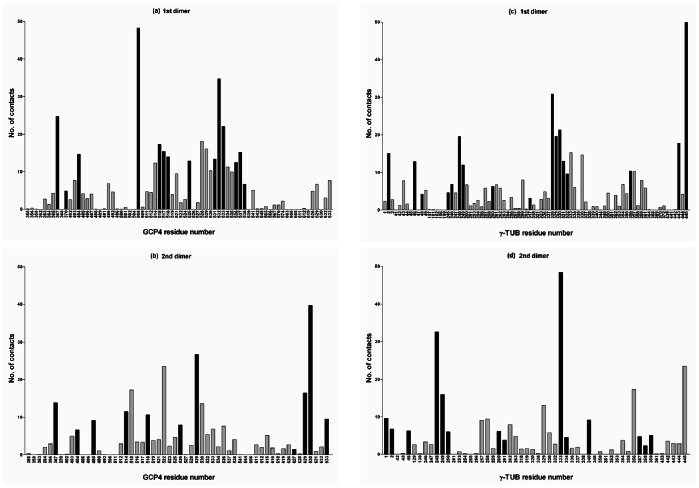
Histograms of contacts between two pairs of GCP4-γ-tubulin (γ-tub) molecules in a tetramer. Persistent contacts (valid in >90% of the MD trajectory frames) are in black. Data are for GCP4 molecules in the two dimers (A and B) and for the γ-tubulin molecules (C and D).

**Figure 3:**
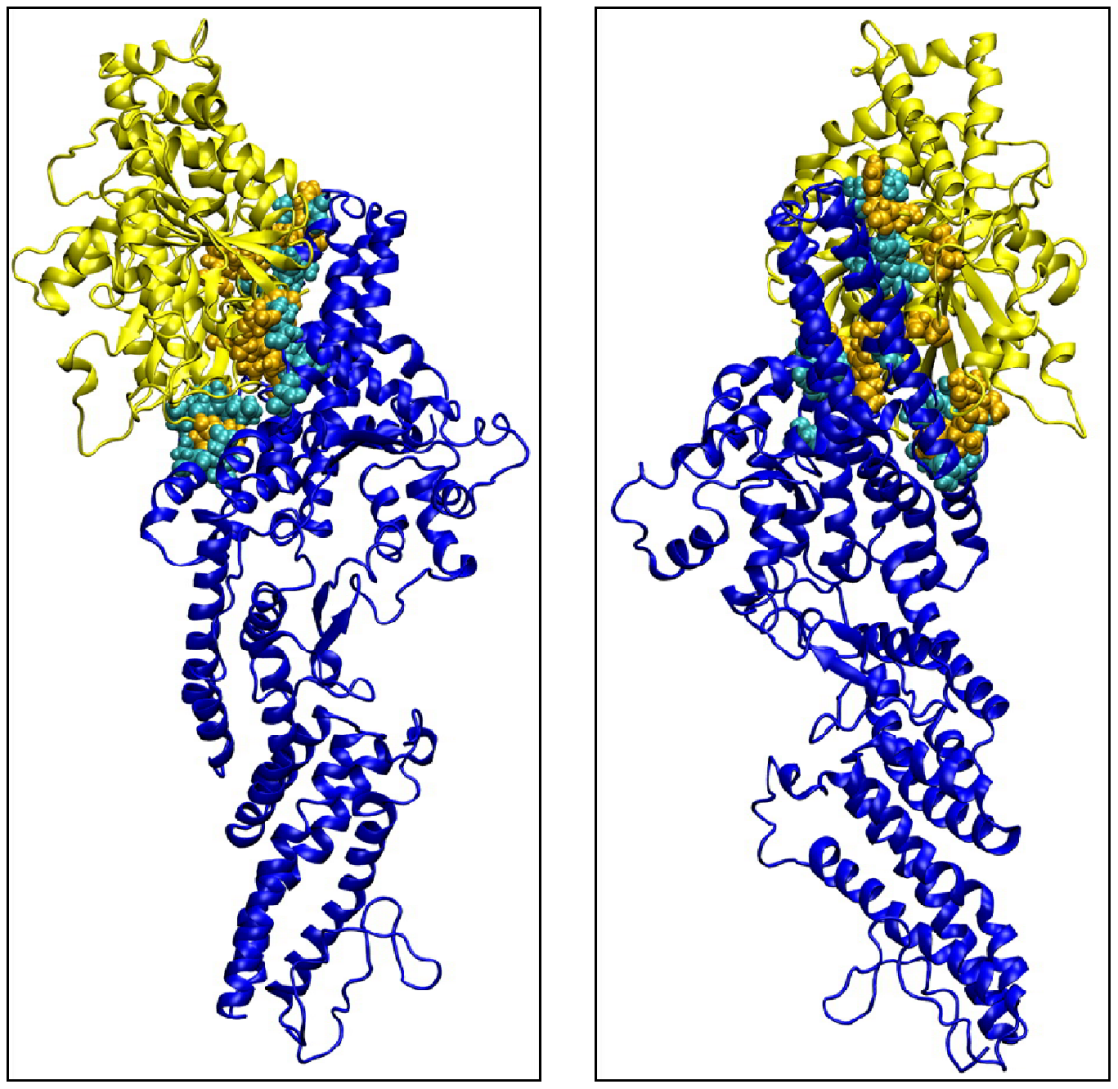
Persistent contacts between GCP4 (blue) and γ-tubulin (yellow) molecules. Two different views of the same dimer are shown. The residues involved in intermolecular contacts are highlighted in cyan (GCP4) and orange (γ-tubulin).

To compute persistent intermolecular contacts at atomic level, we used in-house software, which analyzed trajectory frames (Figures 2 and 3). The program searched for distances between atoms belonging to different molecules below a specified threshold level in each frame. Since data obtained for several thresholds between 3 Å and 5 Å have given similar results, we have used the 3 Å threshold in the final analysis. For each distance found, its fluctuation was monitored across the trajectory to determine the percentage of total simulation time in which the contacts were present. If the percentage exceeded a predetermined threshold (90%), the contacts were reported as persistent. A histogram of interactions between pairs of molecules was created, providing detailed information on intermolecular interactions at atomic level. To double-check the results of our program, we used the Amber utility *ptraj*, which permits finding hydrogen bonds in the studied system. The Amber results represented a subset of our results, because hydrogen bonds are defined not only by the distances but also by the spatial positions of the atoms involved. Within each subset, the results were identical. The interface between the GCP4 and γ-tubulin molecules is described by two sets of amino acids, one for each dimer within the tetramer. The two lists of amino acids are close, but not identical: (i) Glu367, Gln370, Leu404, Gln508, Arg515, Arg517, Asn518, Asp525, Leu531, Gln532, Val533, Leu536, Glu537 and Ser538 for GCP4; Arg2, Arg46, Asp48, Pro245, Gly246, Met248, Asn249, Ile260, Ile317, Ile328, Ala329, Ile330, Leu331, Asn332, Gln356, Ile443 and Trp445 for γ-tubulin in the first dimer, and (ii) Glu367, Leu404, Arg485, Gln508, Trp514, Asn518, Asn526, Tyr529, Lys627, Leu629, Ser630 and Arg633 for GCP4 and Pro1, Arg2, Arg46, Met248, Asn249, Asn250, Ile260, Pro261, His333, Lys334, Arg340, Val357, Ala358 and Leu359 for γ-tubulin in the second dimer. As can be seen in Figure 2, the contacts in both dimers are not the same. This is mostly due to the fact that the interfacial region is rich in flexible loops, whose extents are different in both dimers. For example, examination of the structure of the two contact regions reveals that the helix composed of 15 residues (328 to 342) in dimer BD is only 8 residues long (335 to 342) in dimer AC. Consequently, the RMSD differences between the atomic positions in the corresponding residues in the interface region are significant. However, the superposition of individual molecules (GCP4-A and GCP4-B, as well as γ-tubulin-C and γ-tubulin-D) does not indicate significant deviations (which amount to 6 Å for GCP4 and 3 Å for γ-tubulin).

### Library Description

The fragment library was prepared from the set of Enamine compounds (www.enamine.net), characterized by physicochemical properties according to the rules of Lipinski [6], [7]. Filters were applied for molecular weight from 200 to 500 Da, logP from −5 to 2, molar refractivity from 40 to 130 m^3^.mol^−1^, number of atoms from 20 to 70 (including H-bond donors [e.g.; OH and NH groups] and H-bond acceptors [e.g.; N and O atoms]), and polar surface area not greater than 200 Å^2^ leading to 500 fragments.

**Figure 4:**
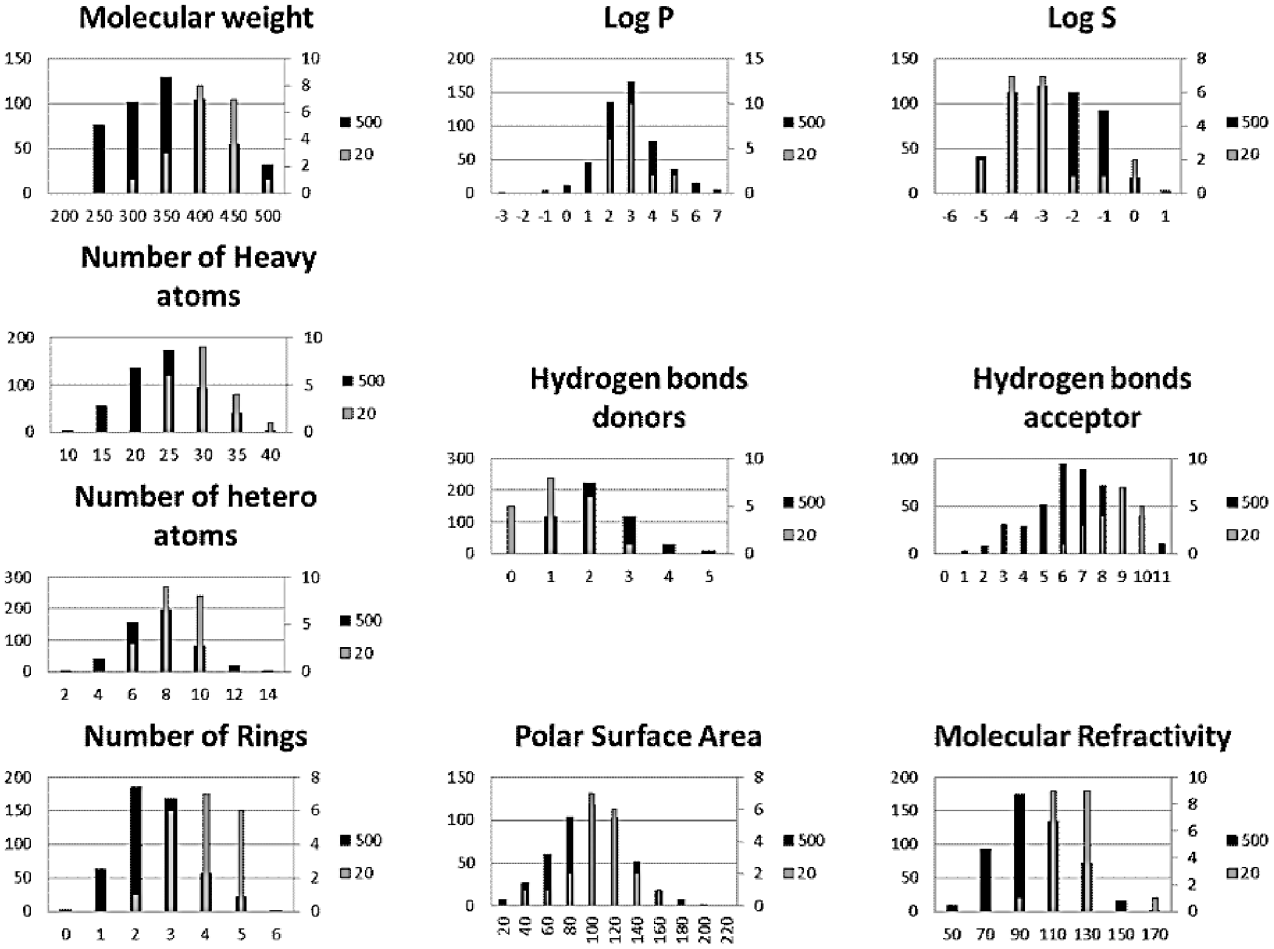
Molecular properties distribution of the fragments of the library used in this study. Molecular weight (MW), LogP, LogS, number of heavy atoms (HAC), hydrogen bonds donors (HD), hydrogen bonds acceptors (HA), number of hetero atoms (nHA), number of rings (NR), polar surface area (PSA) and molar refractivity (MR) of 500-fragment library (black bar chart) and 20-best-fragment library (grey bar chart). The table of the 500 fragments selected is given in supplementary material Figure S3.

Figure 4 shows the distribution of physicochemical properties such as molecular weight (MW), log P, log S, heavy atoms count (HAC), number of hetero-atoms (nHA), number of rings (NR), hydrogen bond donors (HD), hydrogen bond acceptors (HA), the polar surface area (PSA) and the molar refractivity (MR) for the total fragment library with the following average values: MW of 350 Da, logP of 3, logS of -3, PSA of 100 Å^2^, MR of 90 m^3^.mol^−1^, and the average values of HAC, nHA, NR, HD and HA of 25, 8, 2, 2, 6, respectively.

### Screening of the Fragments

**Figure 5:**
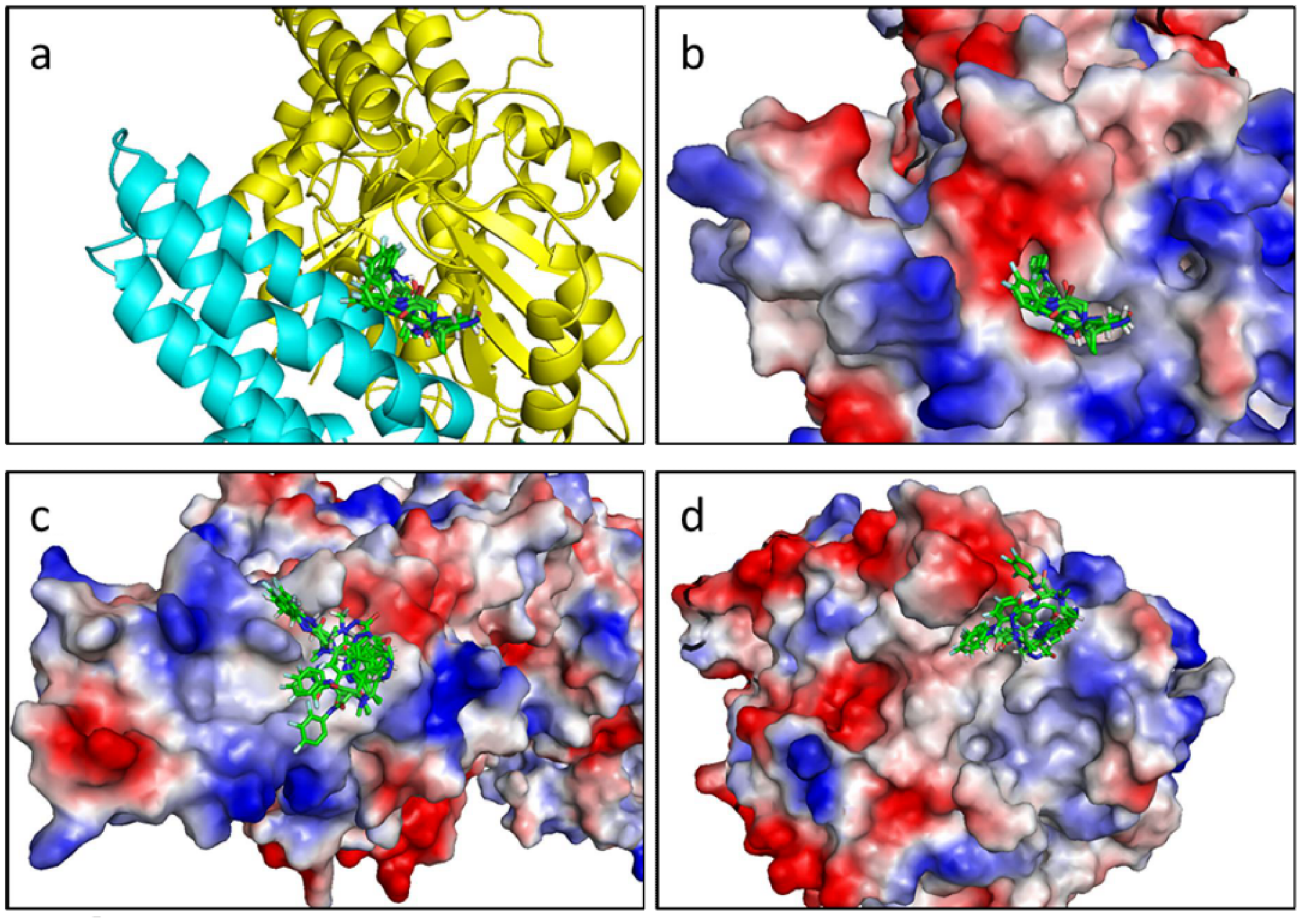
Superimposition of the 10 top positions of the best fragments. a. Cartoon representation of the binding site. b. Electrostatic surface representation of the binding site. c. Top view of the electrostatic surface representation of GCP4. d. Bottom view of the electrostatic surface representation of γ-tubulin.

The 500 members of our library were docked against the full γ-TuSC complex (dimer composed of GCP4 and γ-tubulin) derived from MD using Autodock Vina [32]. Next, the compounds were ranked based on their predicted binding energies and their maximum number of conformations. A large bounding box was used, which encompassed the entire γ-tubulin protein and the C-terminal region of GCP4. The results were processed by the built-in clustering analysis, and the lowest energy conformation from the largest cluster was selected as representative. Binding occurred in the same pocket, localized at the interface between GCP4 and γ-tubulin. It is notable that the same pocket exists on the other side of the γ-TuSC composed of GCP4 chain A and γ-tubulin chain C. The docking results in this area are equivalent to those obtained with chains B and D. Figure 5 shows the superimposition of ten poses of the best fragments in the binding pocket. The pocket, characterized below, was used for a second docking with the same 500-fragment library. The compound rankings were determined for each system. The twenty best molecules were recovered in the first thirty fragments of the 500-fragment library.

### Binding Site Description

Using our 3D γ-TuSC model obtained from molecular dynamics simulations and according to the docking results, we focused our interest on the interface between the two proteins. All the molecules bind the complex in the same region localized on this interface, which allowed us to identify a pocket delineated by the two partners upon binding. This pocket only exists when GCP4 and γ-tubulin associate together. Interestingly, we showed that the region of GCP4 involved in the interaction with γ-tubulin is located on the C-terminal part of the protein between amino acids 349 and 637 (Figure S1. blue background). In regard to γ-tubulin, the C-terminal part is also involved in the interaction (Figure S1. green background). This is in excellent agreement with previous results based on fitting the crystal structure of GCP4 within the cryo-EM density map of γ-TuSC [18].

**Figure 6:**
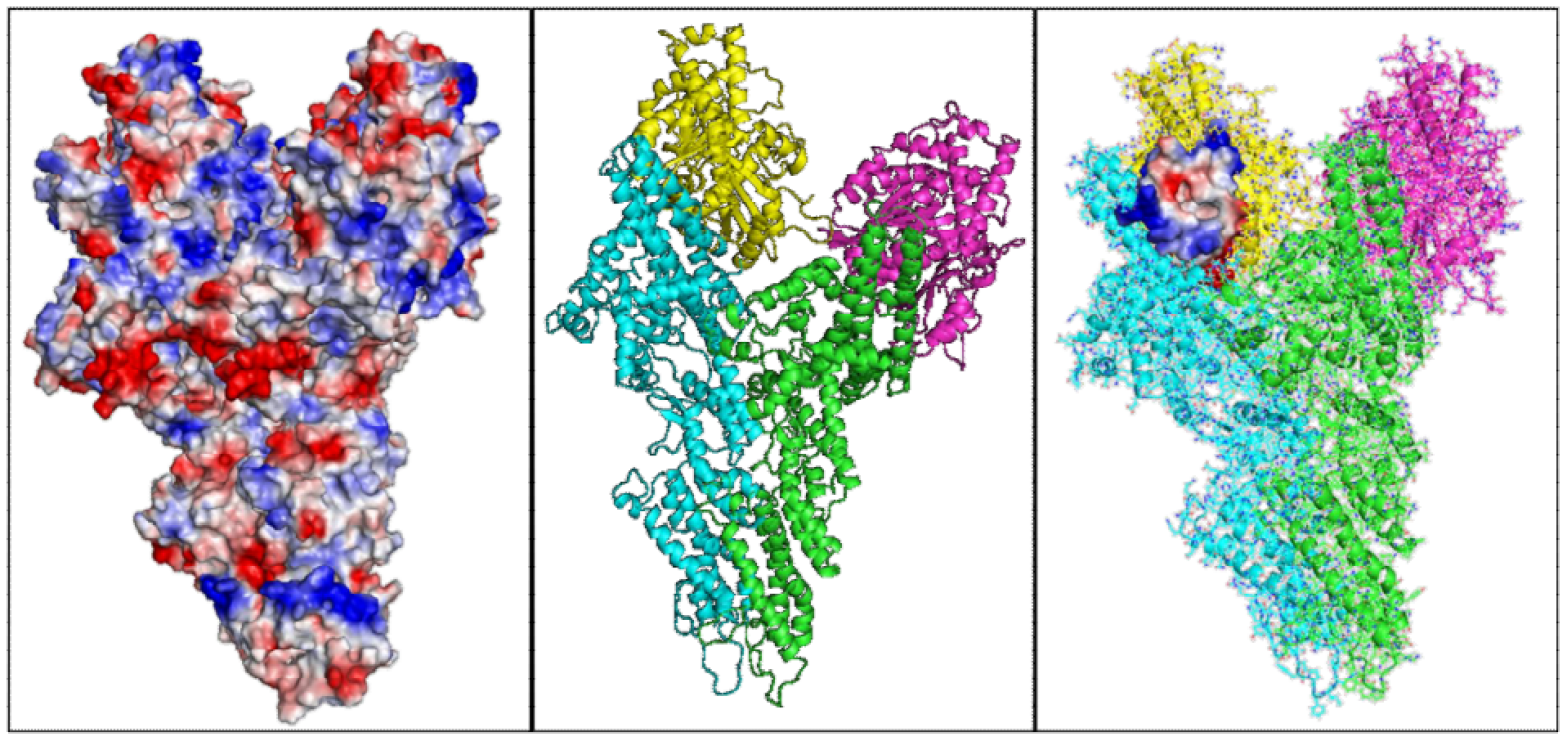
Binding site location. Electrostatic Surface (E.S.) representation of the γ-TuSC (left). Cartoon representation of the γ-TuSC (center) and E.S. representation of the binding pocket located within the γ-TuSC (right).

The pocket formed at the interface between γ-tubulin and GCP4 is composed of residues 246–264, 315–319, 337, 341, 347–358, 379–383, and 442–446 for γ-tubulin and 515–536 and 617–632 for GCP4 (Figure 6). All the lowest-energy positions of ligands obtained from virtual docking of the fragments to γ-TuSC are located within the pocket. Consequently, the refined screening procedures were focused on this binding pocket. By comparing the prediction of the binding site performed with Q-site Finder [37], we found that the best fragments were perfectly aligned with the predictive binding site (Figure S2).

### Experimental Analysis by Differential Scanning Fluorimetry (DSF)

**Figure 7:**
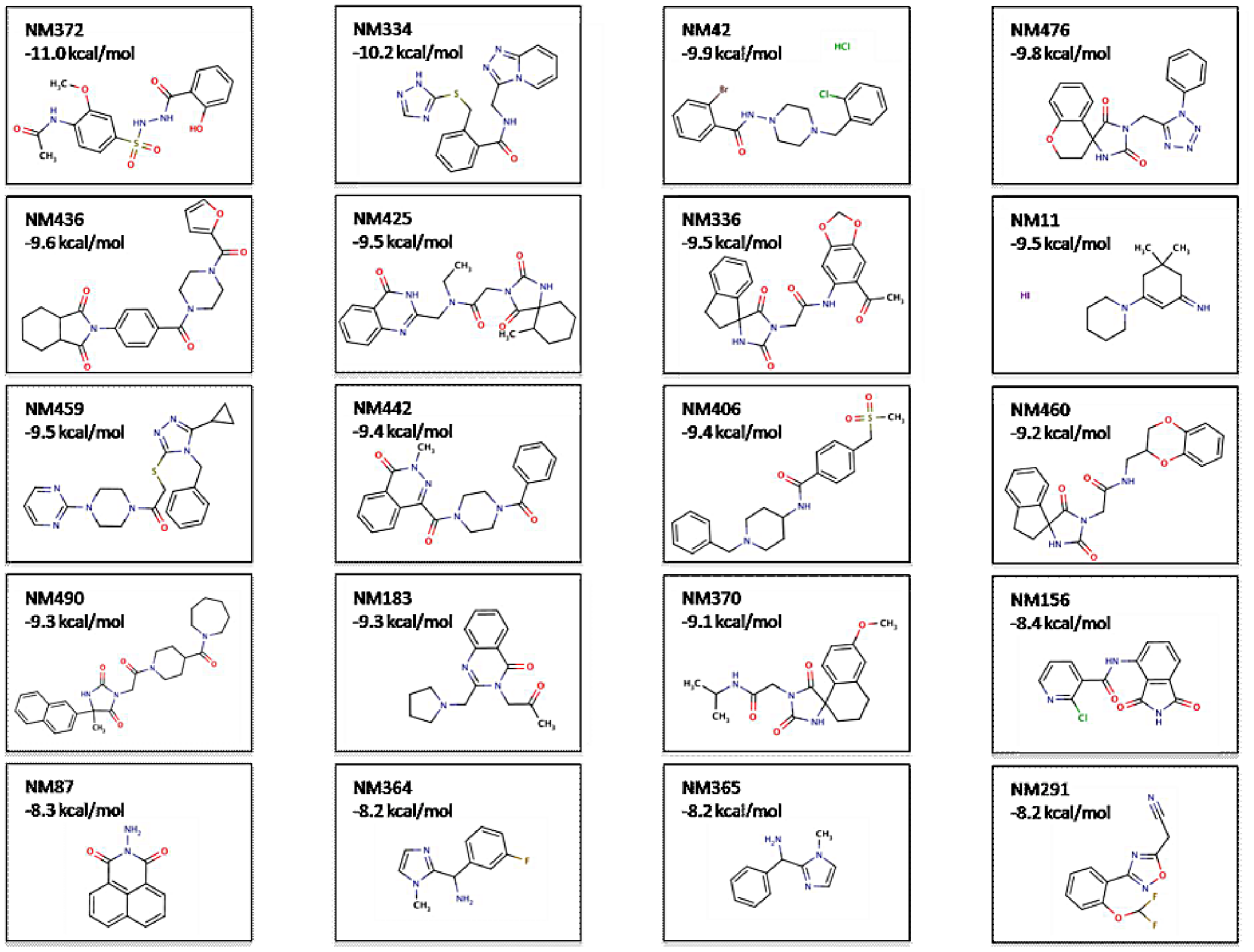
20 best fragments from the virtual screening on the binding site. Molecules have been drawn with Marvin 5.11.3, 2012, ChemAxon (http://www.chemaxon.com). Carbon atoms, black; oxygen atoms, red; nitrogen atoms, blue; sulfur atoms, gold; chlorine atoms, green; fluorine atoms, light brown; bromine atoms, brown; iodine atoms, violet. The energy values correspond to the docking score.

The twenty best compounds identified virtually after sorting on the number of conformations and the interaction energy (Figure 7), were then challenged in an effort to identify potentials inhibitors of γ-TuSC. A biophysical method, DSF [36], was used to identify compounds which alter the melting temperature (T_m_) of the GCP4-γ-tubulin complex.

**Figure 8:**
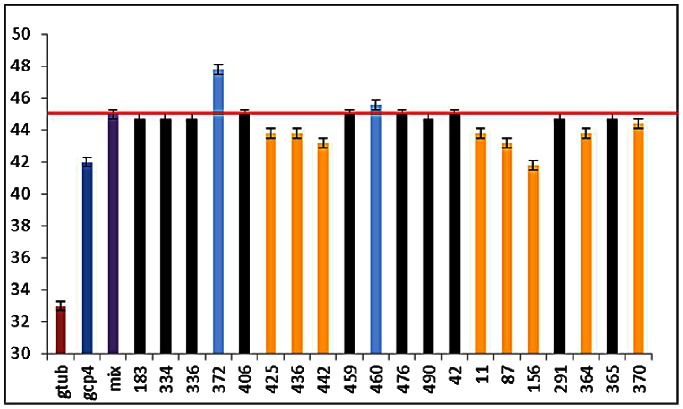
Graphical representation of T_m_ values determined by differential scanning fluorimetry. Melting temperatures (T_m_) of the γ-tubulin-GCP4 complex with the 20 best fragments shown in Figure 7 (500 µM ligand concentration). T_m_ values for γ-tubulin (gtub) and GCP4 (gcp4) are in red and dark blue, respectively, whereas the T_m_ value for the GCP4-γ-tubulin complex (1∶1) is in purple (mix). Black bars correspond to ligands whose addition had no effect on the complex; in orange, ligands showing a significant decrease of the T_m_; in light blue, ligands showing a significant increase of the T_m_. The red horizontal bar represents the T_m_ of the complex. Averages of triplicate data sets are shown. Error bars represent standard errors of the means.

All the fragments were first tested on each protein alone and showed no effect (data not shown). γ-tubulin and GCP4 displayed T_m_ values of 33 and 42°C, respectively whereas a T_m_ of 45°C was found for the stoichiometric complex (Figure 8). This significant shift in melting temperature experimentally confirms that (i) there is formation of the complex composed of the two recombinant proteins *in vitro*, and (ii) that the complex is more stable than each of the two proteins alone. We thus confirmed *in vitro* that GCP4 can bind directly to γ-tubulin. Stable complexes of Flag-tagged γ-tubulin and V5-tagged GCP4 were isolated by immunoprecipitation with anti-Flag affinity beads as previously described [18]. Concerning the binding of the twenty best compounds identified from virtual screening of our library, ten of them (i.e. 50%) had effects on the T_m_ of the complex. Figure S4 shows the ^1^H NMR spectra and structures of the four compounds producing the highest effect in DSF (ΔTm >2°C). The best hits could be separated in two categories where two compounds induced a stabilization of the complex whereas eight compounds induced a destabilization.

However, the fact that ligand binding affected the melting transition temperature of the complex did not guarantee that binding occurred in the expected binding pocket. In order to confirm this, and further confirm the predictive potency of our virtual docking procedure, we made a GCP4 mutant allowing the interaction with the γ-tubulin but blocking access to the pocket.

**Figure 9:**
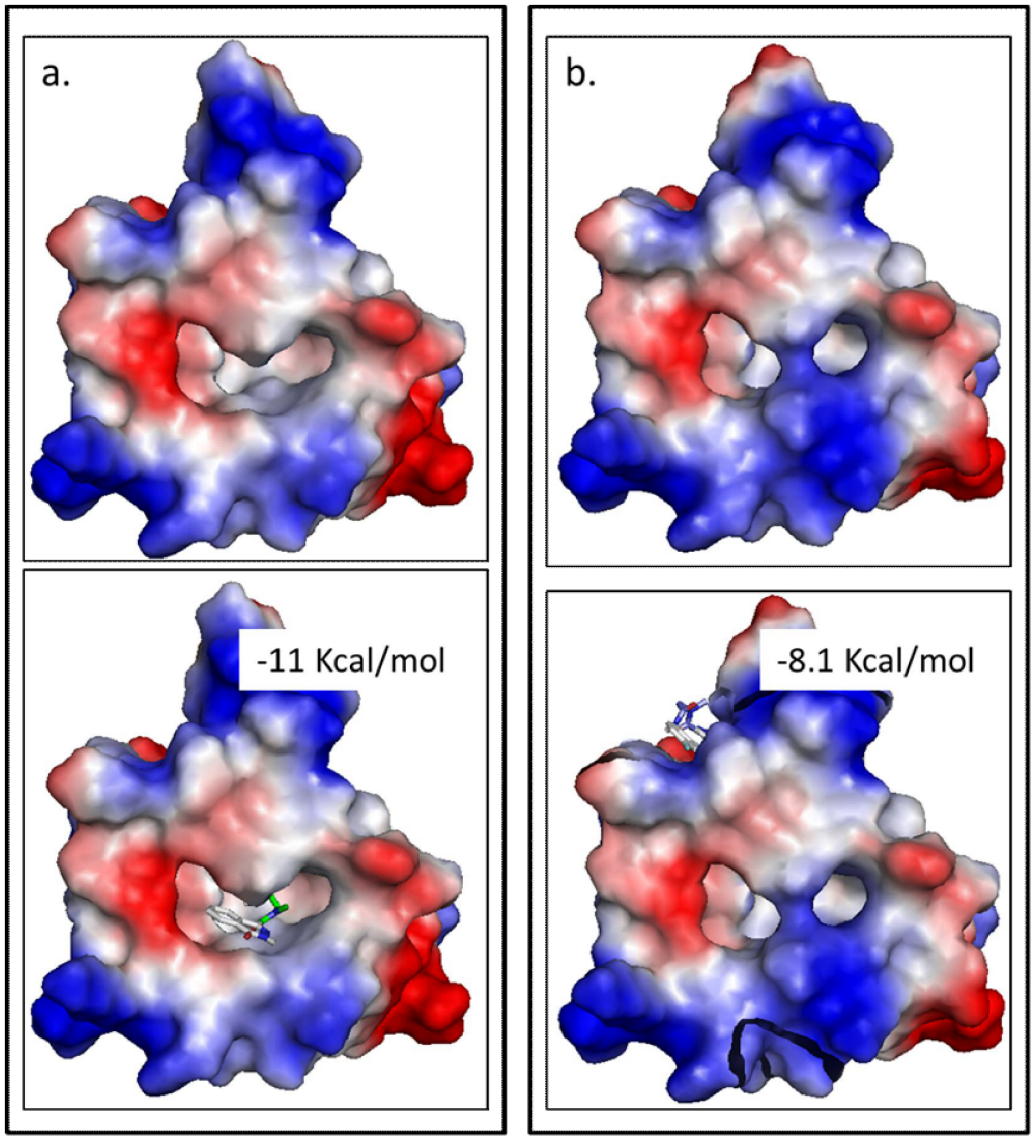
Mutation closing the binding pocket. a. Docking of the best compound, NM372, on the complex composed of GCP4 (WT) and γ-tubulin, without compound (above) and with compound (below). b. Docking of the best compound, NM372, on the complex composed of GCP4 (S623R) and γ-tubulin, without compound (above) and with compound (below). This figure clearly illustrates how the bulky and positively charged arginine side chain blocks the binding pocket.

This mutant was obtained by replacing serine 623 of GCP4 by an arginine and showed a closed state of the binding pocket (Figure 9). Virtual docking with the mutant confirmed that our ligands were unable to bind to the complex in the mutated pocket. The compounds could be found all around the pocket and the binding energies increased from −11.0 kcal/mol for the wild type complex to −8.1 kcal/mol for the mutated complex. To confirm the existence of the binding pocket, expression and purification of the GCP4 S623R protein was performed. Immunoprecipitation assays, performed as previously described [18] revealed that the GCP4 S623R retained the ability to bind directly to γ-tubulin as for the wild type protein.

The GCP4 S623R protein was also used in DSF experiment to validate the specificity of the binding site. The DSF results revealed that GCP4-S623R alone has a T_m_ of 51°C. The increase of T_m_ upon arginine introduction represents a stabilizing effect that has been observed for equivalent mutations in other proteins [38]. In complex with γ-tubulin, the T_m_ of the GCP4 mutant is increased to 54°C, showing a stabilization of the complex to the same extent as for the wild type complex, still confirming the interaction between γ-tubulin and the mutated GCP4.

The addition of the different compounds, even at a ligand concentration of 2 mM, showed no perturbation of the T_m_. Thus, none of our ligands retained their capacity to bind to the complex when the access to the pocket binding site was prevented by an arginine. This in turns shows the specificity of the ligand-complex interaction towards the binding pocket identified by virtual screening.

## Discussion

In this study, we propose a new target to inhibit microtubule assembly. Molecules currently used in cancer chemotherapy, such as paclitaxel and vinca alkaloids, target α/β-tubulin [20], [39], [40]. With the first three-dimensional structure of a γ-tubulin complex protein, GCP4 [18], and the proof of its interaction with γ-tubulin, we found it relevant to target the interface of this complex.

Our strategy was to identify and validate a binding site at the interface between GCP4 and γ-tubulin. We showed the existence of such a binding pocket at the interface of the two proteins and the specificity of the interaction of the best ligands from a designed fragment library in this pocket. Indeed, this binding pocket seemed to be a good target to find potential inhibitors of complex formation. Starting from a compound library of 50,000 fragments filtered according to physical properties and druggability, 500 small compounds were docked against the complex, targeting the binding pocket. The pocket is only created upon complex formation, so when the complex dissociates, the binding pocket no longer exists. We showed that some ligands can also bind to the C-terminal part of GCP4 after complex dissociation, but with a less favorable energy than the one obtained for the binding within the pocket of the complex.

The results obtained by virtual screening were confirmed by biophysical experiments. The twenty best hits identified by docking were used in DSF experiments and we showed that these compounds did not affect the T_m_ of GCP4 and γ-tubulin proteins alone but only induce a T_m_ shift on the GCP4- γ-tubulin complex. 50% of the chemicals induced an effect on this system either through stabilization or destabilization. Ligand-protein binding equilibrium usually leads to the stabilization of the system, and therefore to an increase of the melting temperature as visualized by DSF. Nevertheless, a number of cases have been experimentally observed where equilibrium-binding ligands destabilize proteins, thus decreasing the melting temperature of the system [41] .

Using DSF experiments, we followed the unfolding of the protein. The T_m_ represents a transition where half of the protein concentration is folded and half is unfolded. According to Cimmperman [41], a ligand may bind to the native and/or unfolded protein. If the ligand binds to the unfolded state more strongly than to the native state, then the protein is destabilized by the ligand. On the other hand, if the ligand binds to the native form more strongly than to the unfolded state, then the protein is stabilized by the ligand. This means that some of our compounds bind to the unfolded state better than to the native state. Even if DSF experiments have been used to identify potential ligands, these compounds have to be validated as inhibitors, i) the dissociation constant seems to be around 500 µM but no titration had been done, ii) 500 µM is not enough to be considered as a specific inhibitor and has to be improved, and iii) DSF experiments only show that there is a binding but a structural study has to be performed to confirm the existence of the site and finally an *in vitro* assay has to be done to confirm the inhibition.

To prove the existence of the pocket, we prepared a variant of GCP4 where a single mutation could close the pocket. Indeed, we showed that the pocket could be closed by the single mutation S623R in GCP4 without disturbing its interaction with γ-tubulin. This also proves that the structural conformation of the complex has not been affected. Using virtual and biophysical screening, we showed that no binding could be observed between the previously studied ligands and the complex formed between S623R GCP4 and γ-tubulin.

It is known that defective γ-tubulin complexes lead to abnormalities in microtubule nucleation, and cause aberrant mitotic spindle assembly and cell cycle arrest [9], [10], [42]–[48]. In these previous studies, the assembly of γ-tubulin complexes was usually prevented by depleting essential components of the complex. Since we have shown here that small compounds from a chemical library bind to a hydrophobic pocket at the interface between GCP4 and γ-tubulin, the principal question arises whether such compounds can provoke a biological effect after the complex has already assembled in the cell. Provided that future optimization yields active cell-permeable compounds, we think that this should be possible, since we have successfully demonstrated that several of our compounds lower the melting temperature of the complex and should therefore destabilize existing complexes. Moreover, it is known from expression studies and biochemical analysis that assembly and disassembly of γ-tubulin complexes is a dynamic process [21], [48], and that γ-tubulin complexes inside cells are in a dynamic exchange between an inactive cytoplasmic pool and an active pool bound to microtubule-organizing centers [49], [50]. Consequently, the use of compounds that destabilize γ-tubulin complexes is expected to lower the amount of microtubule-nucleating γ-tubulin in the cell. Such reduced amounts of active γ-tubulin complexes will have an effect of microtubule dynamics, since reduced microtubule nucleation shifts the intracellular equilibrium between microtubule polymer and soluble dimer of α/β-tubulin, and since γ-tubulin complexes may directly affect the stability and dynamic properties of microtubules, in addition to their established role as microtubule nucleators [21], [51]–[55]. As a combined effect, defective γ-tubulin complexes will lead to spindle defects and mitotic arrest, as previously documented by various groups [9], [10], [22], [23], [42]–[48].

Fragment-based lead discovery (FBLD) represents an excellent strategy to screen small and moderately complex molecules up to molecules with higher molecular weight and physical properties closer to drug-like compound [56], [57]. This is because it has been recognized that fragment-like hit molecules can be efficiently developed and optimized toward leads. Several studies have shown that medicinal chemistry optimization of an already drug-like hit or a lead compound can result in a final compound with too high molecular weight and hydrophobicity. In this study, the compounds used were small molecules and displayed an observable effect at 500 µM concentration. The evolution of a lower molecular weight fragment hit represents an attractive alternative approach to optimization as it allows better control of compound properties. Computational chemistry can play an important role both prior to a fragment screen, in producing a target-focused fragment library, and after screening in the evolution of a fragment hit into a drug-like molecule.

In conclusion, we demonstrated that combining molecular dynamics, 3D structure analysis, virtual and biophysical screening was efficient in identifying γ-tubulin complex proteins as a new and very interesting target. Small molecule fragments were identified that bound to the complex composed of GCP4 and γ-tubulin, even though their binding might have been weak. Fragments represent a good tool to detect hot spots and identify new targets. Fragment-based hit identification also represents a powerful technique to build a new specific inhibitor of this binding site by either rational drug design or chemical optimization. Further characterization of the binding pocket and of small molecule inhibitors could represent a promising route for future cancer therapy.

## Supporting Information

Figure S1 **Sequence of the binding site.** γ-tubulin at the top and GCP4 at the bottom. Residues of γ-tubulin and GCP4 involved in the interaction are shown on a green and blue background, respectively. Amino acids in red contribute to the binding pocket.
(DOCX)

Figure S2 **Q-site finder binding site prediction.** a. Cartoon representation of the binding site. GCP4 (green), γ-tubulin (cyan) and predicted binding site (magenta). b. Electrostatic surface representation of the binding site with the best fragment (green stick). c. Cartoon representation of the binding site with superimposition of the fragment in the predicted binding site. d. Electrostatic surface representation of the binding site with the best fragment superimposed to the predicted binding site.
(DOCX)

Figure S3 **Molecular properties distribution of the 500 fragments.** Molecular weight (MW), LogP, LogS, number of heavy atoms (HAC), hydrogen bonds donors (HD), hydrogen bonds acceptors (HA), number of hetero atoms (nHA), number of rings (NR), polar surface area (PSA) and molar refractivity (MR).
(DOCX)

Figure S4 **NMR spectra of the best four hits.** The effects of the best hits from docking on the melting temperature T_m_ of the complex are shown in Fig. 8 of the paper. This figure presents the NMR spectra for a subset of the ligands that had the most pronounced effect on the complex, i.e. whose T_m_ varied by at least 2°C. a. NM372. b. NM156. c. NM87. d. NM442. The 1D spectra have been acquired on a Bruker spectrometer operating at the proton frequency of 600 MHz, using a cryoprobe. Asterisks denote the solvent peaks: DMSO at 2.50 ppm and the residual HDO signal at 3.30 ppm. Molecules shown in insets have been drawn with Marvin 5.11.3, 2012, ChemAxon (http://www.chemaxon.com).
(DOCX)
