# Supplementary material for: Virtual and Biophysical Screening Targeting the γ-Tubulin Complex – A New Target for the Inhibition of Microtubule Nucleation
Source: PLoS One. 2013 May 15;8(5):e63908. doi: 10.1371/journal.pone.0063908 (PMC3655011; doi:10.1371/journal.pone.0063908)
Supplement: Figure S1 — Sequence of the binding site. γ-tubulin at the top and GCP4 at the bottom. Residues of γ-tubulin and GCP4 involved in the interaction are shown on a green and blue background, respectively. Amino acids in red contribute to the binding pocket. (DOCX) [file pone.0063908.s001.docx]

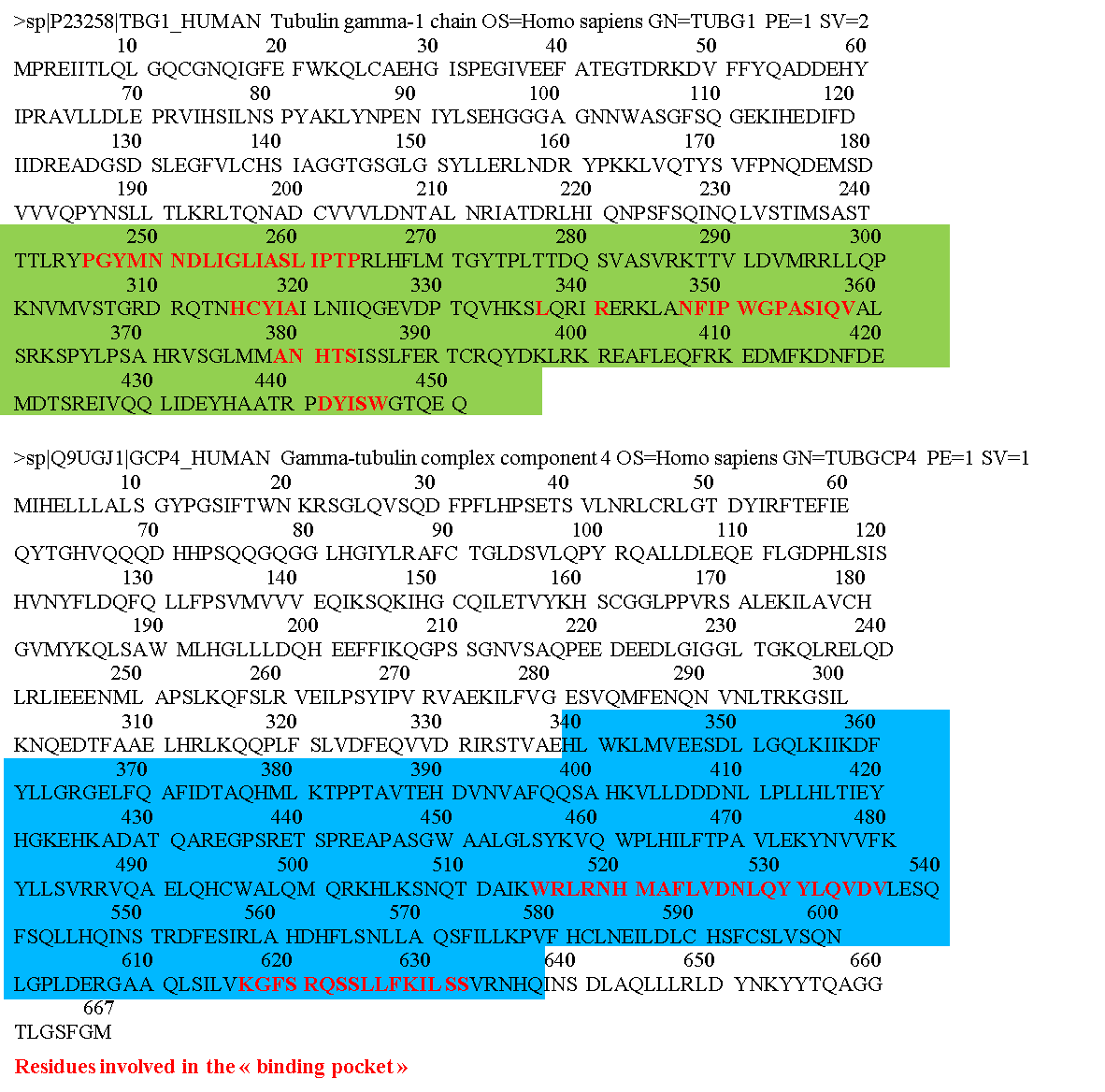


Figure S1 **Sequence of the binding site**. γ-tubulin at the top and GCP4 at the bottom. Residues of γ-tubulin and GCP4 involved in the interaction are shown on a green and blue background, respectively. Amino acids in red contribute to the binding pocket.
