# Supplementary material for: Virtual and Biophysical Screening Targeting the γ-Tubulin Complex – A New Target for the Inhibition of Microtubule Nucleation
Source: PLoS One. 2013 May 15;8(5):e63908. doi: 10.1371/journal.pone.0063908 (PMC3655011; doi:10.1371/journal.pone.0063908)
Supplement: Figure S2 — Q-site finder binding site prediction. a. Cartoon representation of the binding site. GCP4 (green), γ-tubulin (cyan) and predicted binding site (magenta). b. Electrostatic surface representation of the binding site with the best fragment (green stick). c. Cartoon representation of the binding site with superimposition of the fragment in the predicted binding site. d. Electrostatic surface representation of the binding site with the best fragment superimposed to the predicted binding site. (DOCX) [file pone.0063908.s002.docx]

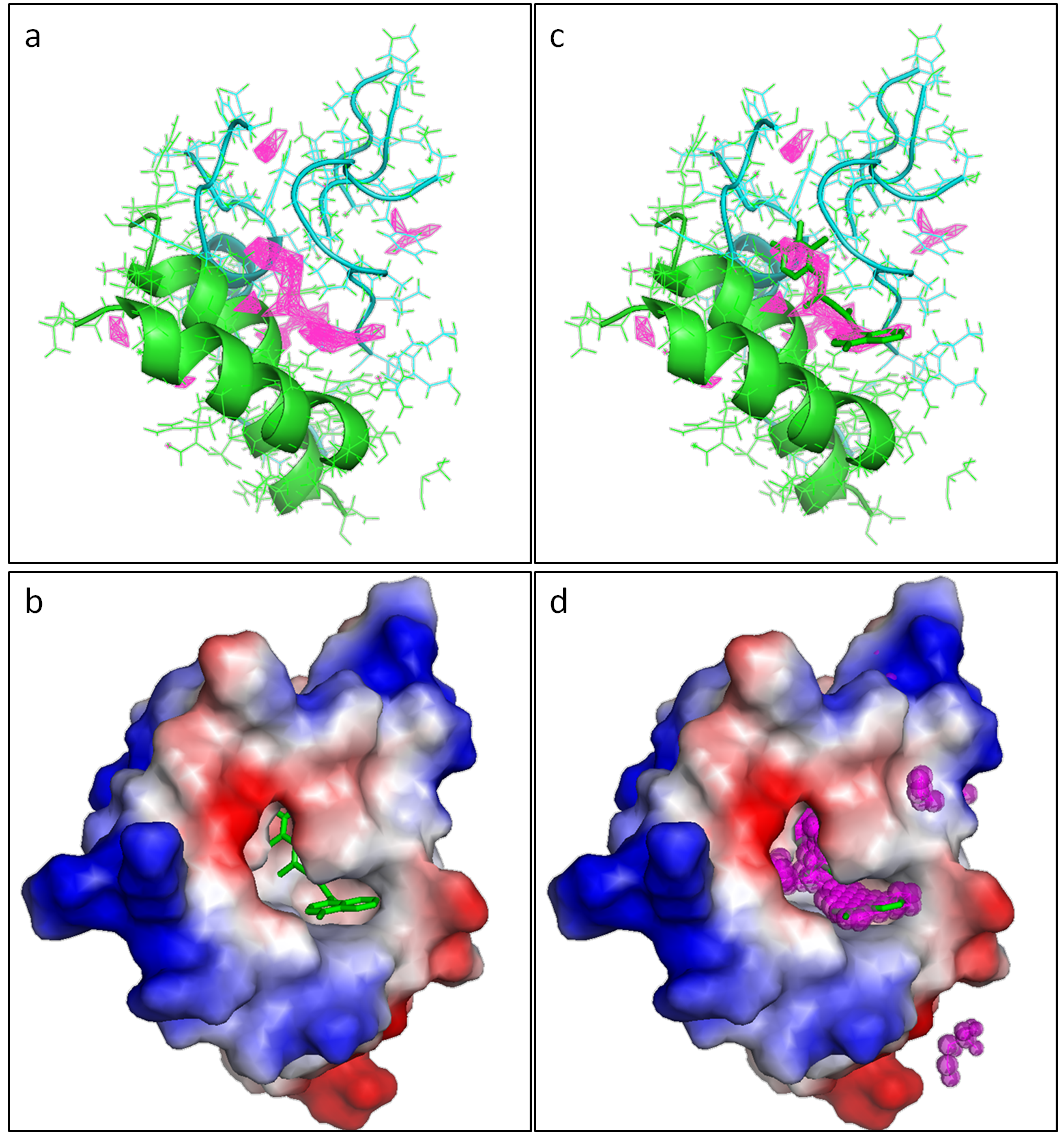


Figure S2 **Q-site finder binding site prediction**

a. Cartoon representation of the binding site. GCP4 (green), γ-tubulin (cyan) and predicted binding site (magenta). b. Electrostatic surface representation of the binding site with the best fragment (green stick). c. Cartoon representation of the binding site with superimposition of the fragment in the predicted binding site. d. Electrostatic surface representation of the binding site with the best fragment superimposed to the predicted binding site.
