# Supplementary material for: Virtual and Biophysical Screening Targeting the γ-Tubulin Complex – A New Target for the Inhibition of Microtubule Nucleation
Source: PLoS One. 2013 May 15;8(5):e63908. doi: 10.1371/journal.pone.0063908 (PMC3655011; doi:10.1371/journal.pone.0063908)
Supplement: Figure S3 — Molecular properties distribution of the 500 fragments. Molecular weight (MW), LogP, LogS, number of heavy atoms (HAC), hydrogen bonds donors (HD), hydrogen bonds acceptors (HA), number of hetero atoms (nHA), number of rings (NR), polar surface area (PSA) and molar refractivity (MR). (DOCX) [file pone.0063908.s003.docx]

| **molecule** | | **MW** | **LOGP** | **LOGS** | **HAC** | **HD** | **HA** | **nHA** | **NR** | **PSA** | **MR** |
| --- | --- | --- | --- | --- | --- | --- | --- | --- | --- | --- | --- |
| 1 | | 247,245 | -0,6293 | -0.792 | 17 | 33 | 6.45 | 7 | 0 | 101,93 | 56,9555 |
| 2 | | 258,768 | 3,8756 | -4 | 16 | 31 | 2.25 | 5 | 2 | 63,21 | 70,8857 |
| 3 | | 226,146 | 1,6975 | -1.592 | 16 | 22 | 2.5 | 9 | 2 | 144,63 | 58,3394 |
| 4 | | 258,314 | 0,8065 | -0.753 | 18 | 40 | 6.5 | 6 | 1 | 76,66 | 65,6354 |
| 5 | | 294,3 | 0,6671 | -1.564 | 21 | 41 | 8 | 6 | 3 | 86,74 | 70,327 |
| 6 | | 307,434 | 3,0073 | -4.963 | 20 | 38 | 5.25 | 6 | 2 | 96,35 | 90,5587 |
| 7 | | 310,369 | 2,0074 | -3.998 | 21 | 40 | 7.9 | 7 | 2 | 100,91 | 81,4894 |
| 8 | | 309,791 | 2,9823 | -3.759 | 21 | 42 | 5 | 6 | 3 | 55,21 | 89,4687 |
| 9 | | 499,324 | 1,099 | -5.664 | 28 | 53 | 5.5 | 4 | 4 | 30,76 | 130,724 |
| 10 | | 359,466 | 3,8326 | -5.167 | 24 | 44 | 5.5 | 7 | 4 | 138,08 | 100,705 |
| 11 | | 334,24 | 4,2318 | -3.948 | 16 | 39 | 2 | 3 | 2 | 27,09 | 85,1767 |
| 12 | | 326,35 | 1,6474 | -2.659 | 24 | 44 | 9 | 7 | 3 | 85,46 | 91,5687 |
| 13 | | 377,304 | -0,1278 | -4.495 | 19 | 48 | 2 | 3 | 3 | 17,07 | 89,0184 |
| 14 | | 327,204 | 0,3215 | -4.476 | 16 | 34 | 0.5 | 2 | 2 | 3,88 | 80,787 |
| 15 | | 459,425 | 5,4263 | -5.586 | 30 | 63 | 5.45 | 7 | 3 | 35,94 | 132,925 |
| 16 | | 492,386 | 4,9598 | -4.639 | 30 | 53 | 5.4 | 9 | 3 | 131,81 | 124,86 |
| 17 | | 368,339 | -0,105 | -1.082 | 26 | 47 | 7.7 | 10 | 3 | 134,63 | 91,9063 |
| 18 | | 310,35 | 2,78288 | -4.666 | 23 | 43 | 6 | 6 | 3 | 83,96 | 86,998 |
| 19 | | 232,067 | -3,6889 | 0.002 | 7 | 12 | 2 | 5 | 0 | 80,29 | 42,7338 |
| 20 | | 447,482 | 5,3979 | -4.76 | 29 | 69 | 6.4 | 6 | 3 | 35,94 | 134,189 |
| 21 | | 405,309 | 5,6573 | -4.043 | 24 | 42 | 4.25 | 6 | 3 | 84,45 | 101,502 |
| 22 | | 251,278 | 1,4571 | -1.737 | 18 | 36 | 5.7 | 5 | 2 | 67,79 | 65,7717 |
| 23 | | 305,371 | 2,7131 | -1.905 | 19 | 35 | 9.25 | 8 | 2 | 106,3 | 70,1007 |
| 24 | | 248,261 | 0,8008 | -1.069 | 17 | 26 | 7 | 7 | 2 | 113,9 | 62,0432 |
| 25 | | 318,25 | -1,0817 | -3.983 | 18 | 41 | 2.45 | 4 | 1 | 29,46 | 79,0462 |
| 26 | | 458,492 | 4,8941 | -5.255 | 33 | 55 | 9.5 | 10 | 5 | 117,07 | 126,064 |
| 27 | | 302,371 | 1,6975 | -3.193 | 22 | 45 | 7.7 | 6 | 2 | 78,99 | 89,5815 |
| 28 | | 375,263 | 4,03538 | -4.58 | 23 | 43 | 5.25 | 6 | 3 | 63,73 | 95,014 |
| 29 | | 343,932 | 3,8061 | -2.848 | 23 | 59 | 5.4 | 4 | 4 | 32,7 | 100,754 |
| 30 | | 219,187 | 2,4107 | -2.872 | 14 | 19 | 4 | 7 | 2 | 65,18 | 45,3787 |
| 31 | | 219,187 | 2,4107 | -2.872 | 14 | 19 | 4 | 7 | 2 | 65,18 | 45,3787 |
| 32 | | 320,255 | 3,5515 | -3.332 | 20 | 43 | 4.45 | 5 | 2 | 32,7 | 89,2238 |
| 33 | | 370,283 | 0,9394 | -5.604 | 23 | 44 | 2.5 | 3 | 3 | 20,95 | 101,079 |
| 34 | | 493,977 | -0,9995 | -5.229 | 34 | 68 | 8.6 | 9 | 4 | 83,45 | 131,28 |
| 35 | | 363,453 | 5,3647 | -5.364 | 27 | 55 | 2.5 | 5 | 5 | 67,15 | 111,861 |
| 36 | | 335,36 | 3,0724 | -5.625 | 25 | 44 | 5 | 7 | 3 | 84,74 | 95,8262 |
| 37 | | 403,273 | 3,79798 | -4.406 | 25 | 46 | 6 | 7 | 4 | 72,96 | 99,392 |
| 38 | | 393,923 | 4,3978 | -4.711 | 27 | 57 | 5.4 | 5 | 3 | 32,7 | 113,025 |
| 39 | | 364,667 | 0,6998 | -4.709 | 21 | 37 | 2 | 5 | 3 | 42,79 | 93,1284 |
| 40 | | 323,328 | 1,9267 | -2.444 | 21 | 33 | 7.5 | 7 | 3 | 80,65 | 75,362 |
| 41 | | 208,24 | 1,4752 | -1.362 | 14 | 23 | 5 | 6 | 2 | 81,51 | 53,443 |
| 42 | | 445,181 | 4,6338 | -4.398 | 25 | 46 | 6 | 7 | 3 | 35,58 | 114,336 |
| 43 | | 214,242 | 1,3512 | -2.579 | 14 | 24 | 6 | 6 | 1 | 96,53 | 52,5132 |
| 44 | | 291,344 | 1,4148 | -1.804 | 18 | 32 | 8.2 | 8 | 2 | 120,53 | 69,1608 |
| 45 | | 291,344 | 1,4148 | -1.804 | 18 | 32 | 8.2 | 8 | 2 | 120,53 | 69,1608 |
| 46 | | 301,303 | 2,3409 | -2.822 | 20 | 34 | 8.5 | 6 | 3 | 106,92 | 83,7059 |
| 47 | | 327,401 | 2,8001 | -4.769 | 23 | 42 | 5.75 | 6 | 3 | 84,27 | 92,9337 |
| 48 | | 469,328 | -0,0469 | -5.718 | 30 | 53 | 6.5 | 7 | 4 | 68,51 | 117,029 |
| 49 | | 303,79 | 3,5119 | -4.153 | 21 | 40 | 4.5 | 6 | 3 | 55,63 | 86,9567 |
| 50 | | 232,987 | -1,4366 | -1.065 | 16 | 27 | 4.2 | 10 | 1 | 144,56 | 55,6562 |
| 51 | | 389,942 | 5,2278 | -4.429 | 26 | 51 | 5.5 | 6 | 3 | 73,47 | 111,948 |
| 52 | | 345,443 | 2,8958 | -4.992 | 23 | 40 | 7.25 | 8 | 3 | 124,21 | 97,8524 |
| 53 | | 477,924 | 6,3858 | -5.043 | 32 | 62 | 5.25 | 10 | 3 | 98,28 | 135,097 |
| 54 | | 413,3 | -0,2483 | -5.346 | 22 | 47 | 5 | 6 | 3 | 52,85 | 104,72 |
| 55 | | 324,259 | 3,9932 | -2.441 | 19 | 42 | 3.5 | 4 | 3 | 20,54 | 88,543 |
| 56 | | 221,133 | 0,9517 | -2.209 | 15 | 21 | 5 | 7 | 1 | 59,3 | 43,5448 |
| 57 | | 221,133 | 0,9517 | -2.209 | 15 | 21 | 5 | 7 | 1 | 59,3 | 43,5448 |
| 58 | | 396,994 | -0,7271 | -3.732 | 13 | 22 | 0.5 | 3 | 2 | 3,88 | 74,276 |
| 59 | | 295,293 | 1,9983 | -3.689 | 22 | 37 | 5 | 6 | 3 | 87,71 | 84,449 |
| 60 | | 392,448 | 3,2497 | -1.502 | 27 | 49 | 3.5 | 9 | 3 | 113,3 | 99,7528 |
| 61 | | 239,291 | 2,5716 | -2.381 | 16 | 30 | 5.75 | 5 | 2 | 63,78 | 63,0367 |
| 62 | | 427,365 | 4,443 | -2.411 | 28 | 57 | 6.95 | 7 | 4 | 49,08 | 125,268 |
| 63 | | 310,372 | 1,0419 | -2.728 | 21 | 39 | 7.2 | 8 | 2 | 119,14 | 86,7975 |
| 64 | | 327,378 | 2,7783 | -4.812 | 24 | 46 | 6.5 | 6 | 2 | 81,18 | 91,6572 |
| 65 | | 456,945 | 3,6851 | -5.114 | 31 | 55 | 8.75 | 9 | 5 | 95,39 | 131,471 |
| 66 | | 349,226 | 2,8266 | -3.531 | 21 | 39 | 5 | 6 | 3 | 61,12 | 91,0554 |
| 67 | | 307,252 | 1,9317 | -4.122 | 22 | 34 | 6.5 | 8 | 2 | 81,18 | 72,0272 |
| 68 | | 377,236 | 3,1666 | -2.064 | 23 | 41 | 5.75 | 7 | 3 | 86,03 | 94,1046 |
| 69 | | 487,976 | 4,1204 | -4.337 | 34 | 66 | 9.45 | 9 | 4 | 82,15 | 136,433 |
| 70 | | 485,444 | 4,6011 | -5.297 | 32 | 66 | 6.95 | 8 | 3 | 54,4 | 140,511 |
| 71 | | 354,274 | 5,2128 | -4.937 | 23 | 44 | 4.25 | 6 | 2 | 47,86 | 101,739 |
| 72 | | 311,294 | 2,5733 | -0.836 | 22 | 39 | 5 | 10 | 2 | 158,77 | 80,4323 |
| 73 | | 434,361 | 4,3263 | -3.717 | 28 | 57 | 8 | 10 | 3 | 84,64 | 127,182 |
| 74 | | 302,276 | 2,0362 | -4.044 | 20 | 30 | 5.5 | 9 | 2 | 85,11 | 66,7127 |
| 75 | | 272,771 | 3,6187 | -3.506 | 18 | 38 | 4 | 5 | 1 | 42,85 | 77,1097 |
| 76 | | 444,956 | 4,9326 | -4.876 | 32 | 60 | 7 | 6 | 5 | 49,33 | 137,356 |
| 77 | | 303,383 | 1,6978 | -3.249 | 21 | 40 | 7.2 | 7 | 3 | 101,29 | 87,3574 |
| 78 | | 380,935 | 4,6266 | -4.977 | 25 | 51 | 6.2 | 7 | 3 | 77,99 | 110,961 |
| 79 | | 431,258 | 4,4844 | -4.524 | 25 | 40 | 4 | 10 | 2 | 144,67 | 97,6076 |
| 80 | | 335,186 | 2,7361 | -2.046 | 21 | 37 | 6.75 | 9 | 2 | 84,89 | 93,456 |
| 81 | | 274,189 | 2,5991 | -1.264 | 17 | 34 | 5 | 5 | 2 | 18,84 | 82,611 |
| 82 | | 308,635 | 3,2525 | -1.892 | 18 | 34 | 5 | 6 | 2 | 18,84 | 87,621 |
| 83 | | 497,54 | 3,1945 | -5.85 | 36 | 69 | 6.5 | 10 | 4 | 138,17 | 139,535 |
| 84 | | 360,676 | 2,4123 | -4.385 | 24 | 33 | 8 | 11 | 2 | 94,07 | 74,9522 |
| 85 | | 244,377 | 1,7077 | -2.784 | 15 | 32 | 4 | 5 | 2 | 92,06 | 71,1507 |
| 86 | | 201,677 | 2,6643 | -2.208 | 12 | 20 | 3 | 5 | 1 | 82,17 | 54,0578 |
| 87 | | 212,204 | 1,2477 | -2.222 | 16 | 26 | 3.5 | 4 | 3 | 65,09 | 63,3124 |
| 88 | | 367,398 | 2,8269 | -4.965 | 27 | 50 | 7.25 | 7 | 3 | 74,49 | 103,455 |
| 89 | | 315,281 | 1,0694 | -1.668 | 23 | 38 | 8.5 | 8 | 3 | 99,64 | 76,4772 |
| 90 | | 311,378 | 2,9963 | -4.891 | 23 | 46 | 5.75 | 5 | 3 | 56,03 | 92,0167 |
| 91 | | 355,817 | 2,9219 | -4.092 | 24 | 46 | 6.75 | 8 | 2 | 74,61 | 92,0612 |
| 92 | | 325,34 | 1,0569 | -2.68 | 22 | 38 | 4.2 | 9 | 3 | 141,09 | 83,3796 |
| 93 | | 383,398 | 2,7082 | -4.152 | 28 | 50 | 4.2 | 8 | 3 | 132,64 | 105,134 |
| 94 | | 383,326 | -0,4435 | -5.548 | 21 | 38 | 1.25 | 6 | 4 | 80,02 | 101,772 |
| 95 | | 351,312 | 2,6814 | -0.938 | 22 | 49 | 7.4 | 6 | 2 | 35,94 | 102,356 |
| 96 | | 428,396 | 4,3738 | -4.736 | 28 | 59 | 5.95 | 7 | 3 | 48,83 | 125,796 |
| 97 | | 393,391 | 3,8411 | -1.798 | 25 | 58 | 6.45 | 6 | 2 | 35,94 | 117,26 |
| 98 | | 265,27 | 1,4189 | -3.264 | 20 | 33 | 5.5 | 6 | 3 | 92,73 | 77,9756 |
| 99 | | 265,27 | 1,4189 | -3.264 | 20 | 33 | 5.5 | 6 | 3 | 92,73 | 77,9756 |
| 100 | | 351,735 | 2,6861 | -4.656 | 22 | 32 | 6.5 | 11 | 2 | 98 | 74,6457 |
| 101 | | 372,888 | 4,2566 | -3.499 | 26 | 53 | 5.75 | 5 | 4 | 32,78 | 113,692 |
| 102 | | 261,75 | 4,1011 | -3.441 | 18 | 34 | 3.5 | 4 | 2 | 37,28 | 78,4527 |
| 103 | | 431,258 | 4,4844 | -4.456 | 25 | 40 | 4 | 10 | 2 | 144,67 | 97,6076 |
| 104 | | 290,813 | 2,0497 | -1.301 | 18 | 37 | 6 | 7 | 2 | 76,32 | 78,516 |
| 105 | | 299,836 | 3,1784 | -2.63 | 20 | 46 | 5.4 | 4 | 2 | 32,7 | 88,5798 |
| 106 | | 423,491 | 2,3427 | -5.41 | 30 | 54 | 9.75 | 10 | 4 | 124,41 | 116,084 |
| 107 | | 269,77 | 2,0414 | -1.778 | 18 | 38 | 5.25 | 5 | 2 | 39,07 | 84,431 |
| 108 | | 302,183 | 3,3903 | -1.458 | 17 | 34 | 4 | 4 | 2 | 20,31 | 75,3655 |
| 109 | | 314,851 | 4,3139 | -2.896 | 21 | 47 | 4.75 | 5 | 1 | 50,36 | 90,6624 |
| 110 | | 220,225 | 1,4074 | -2.62 | 16 | 29 | 8 | 5 | 2 | 67,43 | 64,3124 |
| 111 | | 220,225 | 1,4074 | -2.62 | 16 | 29 | 8 | 5 | 2 | 67,43 | 64,3124 |
| 112 | | 404,506 | 2,1109 | -4.861 | 27 | 49 | 8.7 | 9 | 3 | 144,33 | 115,949 |
| 113 | | 355,614 | 4,4683 | -4.035 | 20 | 32 | 3.5 | 6 | 2 | 61,69 | 85,544 |
| 114 | | 251,258 | 1,2208 | -1.706 | 17 | 27 | 5.5 | 6 | 2 | 108,77 | 64,546 |
| 115 | | 202,209 | 1,3251 | -2.33 | 15 | 27 | 3.5 | 4 | 3 | 58,2 | 60,6724 |
| 116 | | 202,209 | 1,3251 | -2.33 | 15 | 27 | 3.5 | 4 | 3 | 58,2 | 60,6724 |
| 117 | | 231,338 | 1,8557 | -1.897 | 14 | 27 | 5.5 | 6 | 1 | 108,42 | 58,8257 |
| 118 | | 212,249 | 1,8474 | 0.015 | 15 | 30 | 3.5 | 6 | 1 | 111,56 | 59,4596 |
| 119 | | 243,218 | 2,4862 | -2.584 | 18 | 28 | 2 | 6 | 3 | 95,91 | 70,4677 |
| 120 | | 278,284 | 2,2875 | -2.584 | 19 | 30 | 2 | 7 | 3 | 112,84 | 70,3089 |
| 121 | | 249,262 | 1,6599 | -3.468 | 18 | 34 | 5.25 | 5 | 2 | 64,63 | 65,6457 |
| 122 | | 265,378 | 1,2753 | -4.565 | 18 | 38 | 5 | 6 | 2 | 76,38 | 83,3057 |
| 123 | | 226,726 | 2,7534 | -3.972 | 14 | 26 | 1.5 | 4 | 2 | 49,69 | 67,6667 |
| 124 | | 226,726 | 2,7534 | -3.972 | 14 | 26 | 1.5 | 4 | 2 | 49,69 | 67,6667 |
| 125 | | 252,763 | 2,6769 | -4.443 | 16 | 30 | 1.5 | 4 | 3 | 45,53 | 73,577 |
| 126 | | 363,878 | 4,1225 | -4.726 | 25 | 52 | 4.7 | 5 | 3 | 50,72 | 101,766 |
| 127 | | 302,759 | 3,0097 | -3.207 | 21 | 37 | 4 | 6 | 3 | 70,67 | 82,9224 |
| 128 | | 494,533 | 3,7119 | -4.673 | 34 | 62 | 5.5 | 11 | 3 | 161,48 | 128,923 |
| 129 | | 496,534 | 1,1984 | -3.888 | 34 | 63 | 4.5 | 13 | 4 | 209,7 | 131,423 |
| 130 | | 297,42 | 3,1081 | -2.834 | 18 | 30 | 5.5 | 7 | 2 | 147,71 | 75,5524 |
| 131 | | 277,728 | 2,1224 | -1.984 | 17 | 29 | 7.5 | 8 | 1 | 100,88 | 62,7651 |
| 132 | | 277,728 | 2,1224 | -1.984 | 17 | 29 | 7.5 | 8 | 1 | 100,88 | 62,7651 |
| 133 | | 397,319 | 5,1952 | -5.296 | 25 | 44 | 3.5 | 7 | 3 | 69,42 | 104,884 |
| 134 | | 260,292 | 1,2531 | -2.868 | 19 | 36 | 5 | 6 | 2 | 92,91 | 75,5888 |
| 135 | | 260,292 | 1,2531 | -2.868 | 19 | 36 | 5 | 6 | 2 | 92,91 | 75,5888 |
| 136 | | 423,506 | 3,2669 | -2.854 | 28 | 51 | 9.7 | 10 | 3 | 134,85 | 111,422 |
| 137 | | 225,264 | 2,2593 | -0.541 | 15 | 27 | 5.5 | 5 | 2 | 85,61 | 54,7884 |
| 138 | | 228,632 | 1,0287 | -1.583 | 15 | 24 | 5.5 | 6 | 1 | 68,29 | 53,1422 |
| 139 | | 308,353 | 0,6645 | -2.94 | 21 | 38 | 7.5 | 7 | 2 | 107 | 88,464 |
| 140 | | 308,353 | 0,6645 | -2.94 | 21 | 38 | 7.5 | 7 | 2 | 107 | 88,464 |
| 141 | | 431,509 | 1,7932 | -4.184 | 30 | 57 | 8.7 | 10 | 3 | 154,54 | 119,096 |
| 142 | | 341,313 | 0,5493 | -2.354 | 24 | 43 | 8 | 9 | 1 | 109,39 | 80,7122 |
| 143 | | 319,356 | 0,9719 | -0.934 | 23 | 46 | 7.4 | 7 | 3 | 71,11 | 91,7442 |
| 144 | | 253,278 | 1,3267 | -2.793 | 17 | 29 | 5.75 | 7 | 2 | 103,71 | 63,7026 |
| 145 | | 218,32 | 0,4665 | -0.905 | 14 | 32 | 6.7 | 6 | 1 | 94,64 | 62,4538 |
| 146 | | 301,297 | 0,7598 | -2.786 | 22 | 39 | 6.5 | 7 | 3 | 90,29 | 78,6602 |
| 147 | | 379,381 | 3,7172 | -4.386 | 26 | 43 | 7.75 | 9 | 3 | 81,48 | 93,2867 |
| 148 | | 365,378 | 2,7835 | -3.198 | 26 | 49 | 3.5 | 8 | 2 | 118,23 | 93,4803 |
| 149 | | 337,758 | -0,5618 | -4.621 | 23 | 39 | 4.75 | 8 | 2 | 88,03 | 89,9997 |
| 150 | | 388,803 | 6,3466 | -5.364 | 28 | 44 | 4 | 6 | 5 | 75,78 | 111,834 |
| 151 | | 316,848 | 5,6056 | -5.889 | 21 | 39 | 2 | 4 | 3 | 53,16 | 93,2737 |
| 152 | | 365,338 | 3,1604 | -1.793 | 23 | 52 | 6.45 | 6 | 2 | 35,94 | 107,922 |
| 153 | | 208,118 | -1,5656 | -0.261 | 14 | 18 | 2.5 | 8 | 1 | 82,52 | 37,4999 |
| 154 | | 232,235 | 0,80328 | -1.658 | 17 | 29 | 6.5 | 5 | 1 | 70,4 | 59,9535 |
| 155 | | 322,36 | 2,0079 | -4.083 | 23 | 46 | 8 | 8 | 2 | 104,54 | 91,2854 |
| 156 | | 301,685 | 2,2727 | -3.247 | 21 | 31 | 5.5 | 7 | 3 | 88,16 | 78,8389 |
| 157 | | 329,347 | 2,1462 | -3.382 | 24 | 45 | 7.5 | 6 | 3 | 76,82 | 92,3095 |
| 158 | | 351,227 | 3,3924 | -4.393 | 21 | 34 | 5 | 9 | 2 | 99,01 | 83,4314 |
| 159 | | 266,336 | 2,123 | -3.401 | 19 | 42 | 5.5 | 5 | 2 | 66,48 | 79,4787 |
| 160 | | 357,285 | 1,5523 | -2.936 | 25 | 41 | 6.75 | 10 | 3 | 87,74 | 84,7134 |
| 161 | | 307,775 | 2,24798 | -1.505 | 21 | 40 | 7 | 6 | 3 | 48,73 | 88,868 |
| 162 | | 356,374 | 2,876 | -2.987 | 25 | 41 | 7.5 | 8 | 4 | 95,2 | 89,861 |
| 163 | | 205,213 | 1,2943 | -2.276 | 15 | 27 | 4 | 5 | 2 | 75,43 | 60,6221 |
| 164 | | 205,213 | 1,2943 | -2.276 | 15 | 27 | 4 | 5 | 2 | 75,43 | 60,6221 |
| 165 | | 279,292 | 1,6445 | -3.137 | 20 | 37 | 5.5 | 7 | 1 | 97,39 | 70,7889 |
| 166 | | 326,345 | -0,6797 | -1.133 | 23 | 45 | 6.4 | 8 | 2 | 121,54 | 89,9112 |
| 167 | | 292,332 | 2,5479 | -2.546 | 20 | 34 | 5.5 | 7 | 2 | 99,1 | 75,2534 |
| 168 | | 258,069 | 1,6544 | -2.558 | 14 | 22 | 3.75 | 5 | 1 | 66,4 | 53,717 |
| 169 | | 258,069 | 1,6544 | -2.558 | 14 | 22 | 3.75 | 5 | 1 | 66,4 | 53,717 |
| 170 | | 321,8 | 5,857 | -5.513 | 23 | 42 | 1.5 | 3 | 5 | 26,03 | 96,7 |
| 171 | | 412,414 | 3,7391 | -4.047 | 28 | 48 | 5.5 | 11 | 2 | 163,13 | 102,892 |
| 172 | | 403,858 | 6,2227 | -5.65 | 29 | 51 | 3.25 | 5 | 6 | 40,58 | 114,197 |
| 173 | | 317,366 | 2,1335 | -2.893 | 22 | 39 | 7 | 8 | 3 | 110,62 | 81,8037 |
| 174 | | 273,716 | 1,4147 | -3.864 | 18 | 35 | 5.25 | 7 | 2 | 78,51 | 72,9154 |
| 175 | | 273,716 | 1,4147 | -3.864 | 18 | 35 | 5.25 | 7 | 2 | 78,51 | 72,9154 |
| 176 | | 228,171 | 2,63058 | -3.707 | 16 | 23 | 4 | 6 | 1 | 52,89 | 50,3127 |
| 177 | | 228,171 | 2,63058 | -3.707 | 16 | 23 | 4 | 6 | 1 | 52,89 | 50,3127 |
| 178 | | 353,398 | 1,8531 | -0.981 | 25 | 43 | 9 | 8 | 4 | 122,98 | 98,1254 |
| 179 | | 495,056 | 6,0147 | -5.133 | 35 | 73 | 8.5 | 7 | 5 | 67,23 | 145,114 |
| 180 | | 316,44 | 3,0846 | -2.587 | 20 | 41 | 7 | 7 | 2 | 103,1 | 83,5677 |
| 181 | | 336,409 | 2,4252 | -3.653 | 23 | 44 | 9 | 8 | 2 | 92,68 | 86,4172 |
| 182 | | 395,816 | 1,4485 | -1.739 | 25 | 42 | 8.5 | 12 | 1 | 161,49 | 89,442 |
| 183 | | 285,341 | 1,5192 | -0.708 | 21 | 42 | 8 | 5 | 3 | 55,2 | 85,97 |
| 184 | | 421,535 | 3,1678 | -3.719 | 31 | 64 | 9.25 | 7 | 3 | 71,3 | 124,789 |
| 185 | | 397,424 | 1,8541 | -3.935 | 29 | 54 | 8 | 8 | 3 | 88,18 | 112,903 |
| 186 | | 236,267 | 1,0995 | -1.027 | 17 | 33 | 5 | 5 | 1 | 67,43 | 62,9724 |
| 187 | | 421,411 | 2,4408 | -2.611 | 29 | 54 | 6 | 11 | 1 | 118,97 | 100,115 |
| 188 | | 241,267 | 1,6047 | -1.42 | 16 | 28 | 8.2 | 7 | 2 | 83,81 | 58,0727 |
| 189 | | 241,267 | 1,6047 | -1.42 | 16 | 28 | 8.2 | 7 | 2 | 83,81 | 58,0727 |
| 190 | | 320,34 | 1,3293 | -2.136 | 23 | 44 | 4.5 | 7 | 3 | 106,94 | 86,6303 |
| 191 | | 363,388 | 3,0502 | -4.431 | 25 | 43 | 3 | 9 | 3 | 153,79 | 93,6433 |
| 192 | | 225,264 | 1,7355 | -1.118 | 15 | 27 | 4.5 | 5 | 2 | 86,85 | 56,7474 |
| 193 | | 325,359 | 1,7774 | -4.486 | 22 | 39 | 6.5 | 8 | 2 | 103,81 | 89,3484 |
| 194 | | 351,447 | 3,5899 | -4.372 | 23 | 41 | 9 | 9 | 2 | 136,48 | 93,4651 |
| 195 | | 432,553 | 4,3777 | -3.738 | 31 | 68 | 4.5 | 7 | 3 | 106,94 | 120,454 |
| 196 | | 401,456 | 1,4715 | -0.586 | 29 | 57 | 7 | 8 | 3 | 101,39 | 118,784 |
| 197 | | 444,564 | 3,6322 | -3.197 | 32 | 70 | 5 | 7 | 4 | 98,15 | 127,316 |
| 198 | | 385,414 | 2,9577 | -4.145 | 28 | 52 | 8 | 8 | 2 | 105,76 | 105,376 |
| 199 | | 252,742 | 0,5305 | -1.945 | 14 | 23 | 5 | 7 | 1 | 106,8 | 63,8397 |
| 200 | | 252,742 | 0,5305 | -1.945 | 14 | 23 | 5 | 7 | 1 | 106,8 | 63,8397 |
| 201 | | 250,277 | 0,8705 | -3.218 | 17 | 28 | 6 | 7 | 2 | 105,12 | 64,6112 |
| 202 | | 386,442 | 3,6898 | -2.862 | 28 | 54 | 4 | 7 | 2 | 115,73 | 108,299 |
| 203 | | 367,437 | 2,2336 | -2.742 | 26 | 55 | 4.9 | 7 | 2 | 116,09 | 97,8431 |
| 204 | | 242,745 | 3,0356 | -1.941 | 16 | 34 | 4 | 4 | 1 | 41,13 | 70,2264 |
| 205 | | 242,745 | 3,0356 | -1.941 | 16 | 34 | 4 | 4 | 1 | 41,13 | 70,2264 |
| 206 | | 363,679 | 5,2963 | -4.298 | 21 | 39 | 1.5 | 4 | 4 | 27,82 | 96,7924 |
| 207 | | 418,493 | 1,745 | -3.227 | 28 | 48 | 10 | 11 | 3 | 180,51 | 110,206 |
| 208 | | 261,276 | 0,4635 | -2.263 | 19 | 35 | 6 | 6 | 2 | 69,72 | 75,5487 |
| 209 | | 314,405 | 2,2713 | -1.759 | 22 | 42 | 7.5 | 6 | 3 | 79,26 | 89,866 |
| 210 | | 420,245 | 1,2004 | -1.343 | 27 | 46 | 7.5 | 11 | 2 | 127,25 | 106,596 |
| 211 | | 306,208 | 4,3681 | -4.395 | 18 | 31 | 3 | 6 | 2 | 59,59 | 77,261 |
| 212 | | 306,208 | 4,3681 | -4.395 | 18 | 31 | 3 | 6 | 2 | 59,59 | 77,261 |
| 213 | | 329,35 | 1,4336 | -3.016 | 24 | 45 | 7 | 7 | 3 | 92,5 | 92,7703 |
| 214 | | 480,594 | 2,8355 | -3.527 | 34 | 75 | 6.65 | 9 | 3 | 128,56 | 132,946 |
| 215 | | 350,413 | 0,4644 | -1.651 | 25 | 53 | 9.5 | 8 | 3 | 90,03 | 105,868 |
| 216 | | 481,519 | 2,7199 | -2.687 | 33 | 60 | 9.75 | 12 | 2 | 161,93 | 121,85 |
| 217 | | 323,387 | 2,5375 | -2.06 | 23 | 48 | 5.25 | 7 | 1 | 102,68 | 87,1408 |
| 218 | | 346,404 | 1,7046 | -4.203 | 24 | 45 | 8.5 | 8 | 4 | 120,5 | 92,4597 |
| 219 | | 361,267 | 5,7063 | -4.102 | 21 | 32 | 6 | 8 | 3 | 95,68 | 88,7087 |
| 220 | | 447,435 | 1,605 | -3.395 | 32 | 58 | 7.45 | 10 | 3 | 151,62 | 111,983 |
| 221 | | 278,33 | 1,6275 | -3.134 | 19 | 34 | 6.25 | 7 | 2 | 94,34 | 73,3307 |
| 222 | | 249,223 | 0,8319 | -1.594 | 18 | 30 | 5.5 | 7 | 2 | 93,34 | 62,5494 |
| 223 | | 327,373 | 1,5075 | -2.173 | 23 | 47 | 3.95 | 7 | 1 | 116,09 | 86,2951 |
| 224 | | 286,324 | 1,5887 | -0.532 | 20 | 41 | 3 | 7 | 1 | 129,72 | 73,3837 |
| 225 | | 268,312 | 2,2057 | -1.573 | 17 | 27 | 6.5 | 7 | 3 | 118,76 | 62,924 |
| 226 | | 460,305 | 3,0293 | -4.541 | 30 | 53 | 5.2 | 10 | 2 | 116,53 | 112,49 |
| 227 | | 290,357 | 1,9899 | -1.776 | 21 | 44 | 6.25 | 5 | 2 | 72,63 | 84,1604 |
| 228 | | 233,22 | 1,48078 | -3.444 | 17 | 28 | 5 | 5 | 1 | 79,55 | 60,503 |
| 229 | | 239,251 | 1,9958 | -1.102 | 16 | 26 | 6.5 | 7 | 2 | 120,45 | 56,2424 |
| 230 | | 313,311 | 0,8503 | -3.265 | 23 | 40 | 8.75 | 8 | 3 | 91,04 | 84,6377 |
| 231 | | 470,556 | 2,3554 | -2.933 | 33 | 71 | 7.4 | 10 | 2 | 146,58 | 127,075 |
| 232 | | 465,739 | 2,6932 | -3.226 | 28 | 45 | 10 | 12 | 3 | 100,96 | 113,147 |
| 233 | | 393,436 | 2,5927 | -4.641 | 27 | 49 | 7.2 | 10 | 3 | 123,44 | 97,4114 |
| 234 | | 331,646 | 3,7368 | -1.298 | 18 | 31 | 6 | 8 | 2 | 57,79 | 82,3557 |
| 235 | | 331,646 | 3,7368 | -1.298 | 18 | 31 | 6 | 8 | 2 | 57,79 | 82,3557 |
| 236 | | 234,683 | 2,6996 | -1.673 | 13 | 17 | 6 | 7 | 2 | 96,54 | 50,498 |
| 237 | | 234,683 | 2,6996 | -1.673 | 13 | 17 | 6 | 7 | 2 | 96,54 | 50,498 |
| 238 | | 264,234 | 1,2581 | -3.134 | 19 | 32 | 6 | 7 | 2 | 90,54 | 65,6397 |
| 239 | | 352,425 | 1,4168 | -1.417 | 25 | 54 | 9.4 | 7 | 2 | 94,25 | 97,5062 |
| 240 | | 204,182 | 0,4811 | -1.45 | 15 | 24 | 6 | 5 | 2 | 72,19 | 53,8438 |
| 241 | | 204,182 | 0,4811 | -1.45 | 15 | 24 | 6 | 5 | 2 | 72,19 | 53,8438 |
| 242 | | 401,494 | 1,8938 | -2.687 | 28 | 64 | 6.6 | 8 | 3 | 125,32 | 103,183 |
| 243 | | 362,334 | 1,9158 | -2.891 | 26 | 45 | 9 | 9 | 2 | 116,1 | 89,8014 |
| 244 | | 346,275 | 2,9796 | -5.19 | 21 | 39 | 4.2 | 7 | 3 | 71,28 | 98,3037 |
| 245 | | 309,285 | 1,6791 | -2.772 | 21 | 39 | 6 | 9 | 1 | 78,51 | 75,2314 |
| 246 | | 236,673 | 2,7121 | -1.191 | 14 | 23 | 5.5 | 6 | 1 | 60,98 | 52,509 |
| 247 | | 456,849 | 2,7954 | -3.053 | 31 | 53 | 6 | 11 | 2 | 125,4 | 110,975 |
| 248 | | 466,935 | 3,7245 | -4.126 | 31 | 55 | 6.4 | 10 | 3 | 157,22 | 118,438 |
| 249 | | 484,95 | 2,0722 | -4.003 | 32 | 58 | 8.4 | 11 | 3 | 155,85 | 127,403 |
| 250 | | 465,54 | 1,8114 | -1.204 | 33 | 69 | 7.45 | 10 | 3 | 137,43 | 125,172 |
| 251 | | 301,221 | 1,5354 | -3.927 | 21 | 32 | 6 | 9 | 2 | 78,51 | 72,3804 |
| 252 | | 267,324 | 0,7354 | -1.166 | 19 | 42 | 7.2 | 6 | 3 | 61,88 | 80,5137 |
| 253 | | 260,272 | 1,9399 | -2.912 | 18 | 29 | 5.5 | 7 | 4 | 107,91 | 67,4494 |
| 254 | | 303,826 | 4,7766 | -4.007 | 21 | 43 | 2.25 | 3 | 2 | 21,26 | 90,9077 |
| 255 | | 303,826 | 4,7766 | -4.007 | 21 | 43 | 2.25 | 3 | 2 | 21,26 | 90,9077 |
| 256 | | 396,463 | 2,6176 | -4.414 | 28 | 51 | 6.5 | 8 | 4 | 113,75 | 114,576 |
| 257 | | 205,236 | 1,8634 | -2.038 | 14 | 22 | 5.5 | 5 | 2 | 83,12 | 54,1232 |
| 258 | | 415,42 | 4,9307 | -4.526 | 26 | 54 | 5.2 | 6 | 3 | 54,51 | 124,554 |
| 259 | | 324,632 | 6,319 | -5.999 | 20 | 33 | 1 | 4 | 3 | 12,89 | 89,13 |
| 260 | | 439,461 | 1,5626 | -3.933 | 32 | 60 | 9.7 | 9 | 4 | 97,41 | 125,403 |
| 261 | | 340,871 | 2,8346 | -5.191 | 22 | 43 | 5 | 7 | 2 | 91,15 | 103,828 |
| 262 | | 303,399 | 2,4588 | -1.715 | 22 | 48 | 7 | 5 | 2 | 75,43 | 92,0991 |
| 263 | | 448,897 | 3,6008 | -5.801 | 31 | 57 | 4.9 | 9 | 3 | 121,88 | 117,17 |
| 264 | | 257,349 | 2,5078 | -2.436 | 17 | 37 | 7 | 5 | 2 | 71,62 | 66,8017 |
| 265 | | 306,852 | 3,9236 | -2.245 | 19 | 41 | 6.5 | 6 | 1 | 57,79 | 81,1997 |
| 266 | | 369,929 | 4,3652 | -1.071 | 25 | 58 | 6.5 | 6 | 3 | 57,51 | 106,323 |
| 267 | | 299,863 | 5,2399 | -4.696 | 19 | 41 | 2 | 5 | 2 | 72,08 | 87,3314 |
| 268 | | 299,863 | 5,2399 | -4.696 | 19 | 41 | 2 | 5 | 2 | 72,08 | 87,3314 |
| 269 | | 370,873 | 5,0211 | -4.069 | 26 | 52 | 2.5 | 5 | 5 | 46,28 | 109,459 |
| 270 | | 229,635 | 2,2786 | -3.079 | 15 | 24 | 4.5 | 5 | 1 | 46,17 | 55,7032 |
| 271 | | 229,635 | 2,2786 | -3.079 | 15 | 24 | 4.5 | 5 | 1 | 46,17 | 55,7032 |
| 272 | | 269,767 | 3,3206 | -1.861 | 18 | 38 | 4.75 | 4 | 2 | 40,54 | 79,3225 |
| 273 | | 269,767 | 3,3206 | -1.861 | 18 | 38 | 4.75 | 4 | 2 | 40,54 | 79,3225 |
| 274 | | 251,348 | 2,2723 | -2.931 | 17 | 34 | 5 | 5 | 1 | 85,25 | 73,5361 |
| 275 | | 272,306 | 0,3337 | -0.82 | 20 | 38 | 8 | 7 | 3 | 67,15 | 80,421 |
| 276 | | 284,31 | 2,3703 | -2.594 | 21 | 38 | 6.75 | 5 | 2 | 68,29 | 78,0262 |
| 277 | | 292,34 | 1,628 | -2.825 | 19 | 29 | 8 | 9 | 3 | 138,61 | 72,3892 |
| 278 | | 283,325 | 1,4149 | -1.723 | 21 | 40 | 7 | 5 | 3 | 53,51 | 87,6805 |
| 279 | | 372,461 | 1,9315 | -3.287 | 27 | 57 | 9.45 | 7 | 3 | 68,5 | 110,001 |
| 280 | | 261,252 | 1,2403 | -3.898 | 19 | 32 | 4.5 | 6 | 2 | 63,99 | 67,2002 |
| 281 | | 460,563 | 4,0043 | -3.418 | 33 | 71 | 4.75 | 8 | 4 | 124,96 | 124,478 |
| 282 | | 232,664 | 2,3006 | -1.352 | 15 | 27 | 4.7 | 6 | 1 | 77,07 | 58,4789 |
| 283 | | 232,664 | 2,3006 | -1.352 | 15 | 27 | 4.7 | 6 | 1 | 77,07 | 58,4789 |
| 284 | | 267,283 | 2,03938 | -3.464 | 20 | 34 | 5.75 | 5 | 2 | 75,01 | 72,5467 |
| 285 | | 232,278 | 1,7852 | -2.385 | 17 | 34 | 4.25 | 4 | 2 | 58,2 | 64,5199 |
| 286 | | 395,405 | 3,0811 | -4.198 | 27 | 47 | 8.5 | 10 | 3 | 117,1 | 96,0109 |
| 287 | | 217,242 | 2,1323 | -1.49 | 14 | 25 | 6 | 6 | 1 | 87 | 50,4204 |
| 288 | 217,242 | 2,1323 | -1.49 | 14 | 25 | 6 | 6 | 1 | 87 | 50,4204 |  |
| 289 | 375,373 | 2,5686 | -3.214 | 27 | 49 | 3.75 | 8 | 3 | 114,32 | 96,2963 |  |
| 290 | 337,821 | 0,1553 | -4.645 | 22 | 39 | 3.25 | 6 | 3 | 82,56 | 92,003 |  |
| 291 | 251,189 | 2,40408 | -3.546 | 18 | 26 | 5 | 7 | 2 | 71,94 | 55,85 |  |
| 292 | 251,189 | 2,40408 | -3.546 | 18 | 26 | 5 | 7 | 2 | 71,94 | 55,85 |  |
| 293 | 370,626 | -0,5899 | -4.319 | 21 | 34 | 4.5 | 6 | 2 | 47,25 | 84,581 |  |
| 294 | 365,491 | 0,2897 | -4.803 | 16 | 28 | 0.5 | 4 | 2 | 3,88 | 78,746 |  |
| 295 | 301,364 | 2,747 | -2.901 | 21 | 39 | 5.5 | 6 | 4 | 99,16 | 82,1934 |  |
| 296 | 327,373 | 2,1464 | -2.74 | 23 | 47 | 3.95 | 7 | 1 | 105,09 | 85,9003 |  |
| 297 | 327,334 | 4,1529 | -3.648 | 22 | 36 | 7 | 8 | 2 | 93,54 | 79,2744 |  |
| 298 | 300,332 | 2,5376 | -2.965 | 21 | 35 | 6.75 | 6 | 3 | 88,91 | 80,5125 |  |
| 299 | 364,371 | 1,5423 | -3.77 | 26 | 48 | 8.7 | 9 | 2 | 110,42 | 96,3756 |  |
| 300 | 433,477 | 1,3739 | -4.316 | 31 | 61 | 9.2 | 10 | 3 | 111,59 | 119,099 |  |
| 301 | 376,447 | 2,896 | -4.094 | 27 | 55 | 3.2 | 7 | 2 | 100,79 | 103,624 |  |
| 302 | 324,349 | 2,7745 | -4.947 | 22 | 38 | 5.5 | 8 | 3 | 85,11 | 79,2207 |  |
| 303 | 312,365 | 3,5105 | -3.133 | 20 | 33 | 10 | 8 | 2 | 135,11 | 76,0196 |  |
| 304 | 351,785 | 1,1704 | -2.613 | 24 | 44 | 8.2 | 8 | 3 | 78,95 | 97,7487 |  |
| 305 | 384,452 | 3,2298 | -2.233 | 27 | 49 | 8 | 8 | 3 | 115,32 | 102,021 |  |
| 306 | 268,289 | 0,938 | 0.098 | 18 | 31 | 8 | 7 | 2 | 83,14 | 67,1375 |  |
| 307 | 331,406 | 3,1474 | -3.177 | 24 | 51 | 1.5 | 5 | 4 | 86,63 | 91,2093 |  |
| 308 | 293,365 | 2,5445 | -3.319 | 19 | 31 | 7.5 | 7 | 2 | 125,49 | 75,6582 |  |
| 309 | 296,276 | 1,0776 | -1.624 | 21 | 37 | 7.5 | 8 | 1 | 116,95 | 72,7326 |  |
| 310 | 425,481 | 2,4074 | -5.795 | 31 | 61 | 9.25 | 9 | 4 | 105,44 | 123,959 |  |
| 311 | 267,776 | 2,9398 | -1.321 | 16 | 33 | 5 | 7 | 1 | 74,44 | 65,8957 |  |
| 312 | 267,776 | 2,9398 | -1.321 | 16 | 33 | 5 | 7 | 1 | 74,44 | 65,8957 |  |
| 313 | 326,865 | -1,84482 | -2.187 | 22 | 50 | 6 | 6 | 3 | 56,13 | 96,0047 |  |
| 314 | 374,391 | 0,5711 | -3.059 | 27 | 52 | 10 | 9 | 4 | 103,17 | 108,421 |  |
| 315 | 312,32 | 2,8355 | -3.626 | 23 | 41 | 6.7 | 6 | 3 | 77,61 | 83,3328 |  |
| 316 | 243,756 | 3,7308 | -3.401 | 15 | 29 | 2 | 5 | 2 | 72,08 | 68,1034 |  |
| 317 | 243,756 | 3,7308 | -3.401 | 15 | 29 | 2 | 5 | 2 | 72,08 | 68,1034 |  |
| 318 | 254,756 | 2,8241 | -0.851 | 17 | 36 | 4 | 4 | 2 | 41,13 | 74,8194 |  |
| 319 | 254,756 | 2,8241 | -0.851 | 17 | 36 | 4 | 4 | 2 | 41,13 | 74,8194 |  |
| 320 | 343,354 | 1,2358 | -4.319 | 24 | 39 | 5.5 | 7 | 3 | 120,77 | 90,2172 |  |
| 321 | 422,3 | 3,592 | -4.387 | 27 | 52 | 3.95 | 9 | 2 | 116,09 | 103,338 |  |
| 322 | 286,309 | 1,5647 | -2.796 | 20 | 32 | 8 | 7 | 3 | 105,12 | 76,7707 |  |
| 323 | 344,365 | 0,7236 | -4.122 | 25 | 47 | 7.5 | 8 | 3 | 84,74 | 99,4835 |  |
| 324 | 480,488 | 2,8859 | -4.429 | 35 | 64 | 9.5 | 10 | 5 | 93,11 | 135,779 |  |
| 325 | 403,426 | 2,2034 | -3.358 | 29 | 54 | 5.95 | 8 | 2 | 133,16 | 106,079 |  |
| 326 | 360,312 | 2,3664 | -4.076 | 24 | 37 | 7 | 11 | 3 | 103,57 | 77,9037 |  |
| 327 | 300,783 | 5,1778 | -4.893 | 21 | 39 | 2 | 4 | 3 | 30,96 | 89,543 |  |
| 328 | 316,825 | 4,8698 | -5.464 | 22 | 44 | 2.25 | 4 | 3 | 27,05 | 93,36 |  |
| 329 | 220,269 | 1,3412 | -1.453 | 13 | 22 | 8 | 7 | 2 | 120,24 | 47,787 |  |
| 330 | 220,269 | 1,3412 | -1.453 | 13 | 22 | 8 | 7 | 2 | 120,24 | 47,787 |  |
| 331 | 296,589 | 3,4698 | -1.443 | 15 | 29 | 2.5 | 5 | 1 | 44,48 | 66,5714 |  |
| 332 | 296,589 | 3,4698 | -1.443 | 15 | 29 | 2.5 | 5 | 1 | 44,48 | 66,5714 |  |
| 333 | 260,38 | 2,3468 | -2.042 | 16 | 32 | 6 | 7 | 1 | 134,44 | 67,8781 |  |
| 334 | 365,412 | 2,4606 | -2.849 | 26 | 44 | 8.5 | 9 | 4 | 126,16 | 96,6189 |  |
| 335 | 259,304 | 1,7712 | -2.452 | 19 | 37 | 4.5 | 5 | 2 | 74,85 | 74,4044 |  |
| 336 | 421,403 | 2,2896 | -4.741 | 31 | 54 | 8.5 | 9 | 5 | 114,04 | 115,665 |  |
| 337 | 376,561 | 3,3335 | -3.132 | 23 | 47 | 8 | 9 | 1 | 162,82 | 97,4244 |  |
| 338 | 318,409 | 1,0732 | -0.983 | 22 | 51 | 5.2 | 7 | 1 | 99,1 | 88,4603 |  |
| 339 | 389,407 | 1,7508 | -1.395 | 29 | 51 | 10 | 8 | 4 | 111,81 | 110,062 |  |
| 340 | 260,335 | 1,3183 | -2.113 | 19 | 41 | 5.5 | 5 | 3 | 49,33 | 80,413 |  |
| 341 | 395,409 | 2,3615 | -5.204 | 29 | 52 | 8.5 | 8 | 3 | 91,56 | 107,862 |  |
| 342 | 343,38 | 0,2127 | -4.545 | 25 | 48 | 8 | 8 | 3 | 93,57 | 103,059 |  |
| 343 | 303,36 | 1,0001 | -1.932 | 22 | 44 | 6 | 7 | 2 | 77,57 | 90,4504 |  |
| 344 | 356,422 | 3,0221 | -3.886 | 24 | 39 | 7.5 | 8 | 4 | 126,83 | 92,856 |  |
| 345 | 327,404 | 2,5333 | -4.589 | 23 | 43 | 6.5 | 7 | 4 | 101,76 | 90,6867 |  |
| 346 | 363,435 | 2,1482 | -4.294 | 25 | 47 | 9.7 | 9 | 2 | 123,44 | 96,6574 |  |
| 347 | 304,298 | 2,1735 | -2.154 | 22 | 39 | 7 | 7 | 2 | 103,79 | 76,8896 |  |
| 348 | 309,323 | 2,0816 | -3.812 | 23 | 40 | 8 | 7 | 3 | 88,39 | 84,7829 |  |
| 349 | 279,335 | 1,5674 | -2.749 | 20 | 43 | 6 | 6 | 3 | 78,51 | 80,4264 |  |
| 350 | 242,321 | 1,3401 | -2.807 | 15 | 26 | 5 | 7 | 2 | 117,11 | 60,7967 |  |
| 351 | 337,801 | 2,2421 | -3.5 | 23 | 44 | 6.5 | 7 | 2 | 69,72 | 94,8627 |  |
| 352 | 334,347 | 1,1089 | -4.195 | 23 | 38 | 7.5 | 8 | 2 | 122,71 | 83,2302 |  |
| 353 | 335,643 | -0,8651 | -2.94 | 18 | 36 | 2 | 5 | 3 | 3,24 | 84,212 |  |
| 354 | 203,197 | 0,6736 | -2.563 | 15 | 25 | 3.5 | 5 | 2 | 74,85 | 55,3599 |  |
| 355 | 389,919 | 3,446 | -2.69 | 27 | 56 | 5 | 6 | 3 | 37,3 | 119,72 |  |
| 356 | 283,282 | 1,6968 | -1.895 | 21 | 36 | 7 | 6 | 3 | 77,13 | 76,6337 |  |
| 357 | 283,241 | 4,6608 | -2.178 | 15 | 26 | 2 | 6 | 1 | 100,47 | 73,6411 |  |
| 358 | 235,259 | 1,5026 | -1.99 | 16 | 26 | 5 | 5 | 2 | 79,75 | 64,1345 |  |
| 359 | 376,453 | 3,1809 | -2.869 | 25 | 43 | 9.5 | 9 | 3 | 118,32 | 97,5945 |  |
| 360 | 246,353 | 2,1008 | -1.7 | 15 | 29 | 8 | 7 | 1 | 134,44 | 63,0711 |  |
| 361 | 325,388 | 2,7686 | -3.861 | 23 | 40 | 6 | 7 | 3 | 98 | 89,5012 |  |
| 362 | 205,231 | 2,3076 | -1.299 | 15 | 28 | 2.5 | 4 | 2 | 43,84 | 55,6074 |  |
| 363 | 205,231 | 2,3076 | -1.299 | 15 | 28 | 2.5 | 4 | 2 | 43,84 | 55,6074 |  |
| 364 | 205,231 | 2,3076 | -1.46 | 15 | 28 | 2.5 | 4 | 2 | 43,84 | 55,6074 |  |
| 365 | 205,231 | 2,3076 | -1.46 | 15 | 28 | 2.5 | 4 | 2 | 43,84 | 55,6074 |  |
| 366 | 379,434 | 2,2801 | -2.822 | 26 | 49 | 9.75 | 10 | 3 | 138,82 | 97,6611 |  |
| 367 | 207,724 | 2,42148 | -2.126 | 12 | 24 | 3 | 5 | 0 | 73,48 | 56,5547 |  |
| 368 | 354,36 | 2,0514 | -3.608 | 26 | 47 | 9.2 | 8 | 4 | 100,46 | 99,5369 |  |
| 369 | 342,338 | 1,9004 | -2.325 | 22 | 39 | 8.5 | 11 | 1 | 92,68 | 71,8317 |  |
| 370 | 345,393 | 1,9608 | -3.332 | 25 | 50 | 6.75 | 7 | 3 | 87,74 | 99,1024 |  |
| 371 | 423,506 | 3,1673 | -4.996 | 28 | 50 | 7.5 | 10 | 2 | 154,71 | 109,986 |  |
| 372 | 379,388 | 2,9181 | -3.155 | 26 | 44 | 9.25 | 10 | 2 | 142,21 | 92,8756 |  |
| 373 | 399,463 | 2,5708 | -4.652 | 28 | 52 | 9 | 8 | 4 | 109,22 | 111,637 |  |
| 374 | 344,392 | 1,5192 | -4.79 | 24 | 42 | 7 | 9 | 3 | 119,48 | 89,2112 |  |
| 375 | 274,341 | 2,0525 | -1.692 | 19 | 34 | 5.5 | 6 | 2 | 85,11 | 74,8357 |  |
| 376 | 303,379 | 2,1626 | -1.905 | 21 | 40 | 6 | 6 | 3 | 95,86 | 86,8304 |  |
| 377 | 358,392 | 1,4395 | -3.537 | 26 | 50 | 7.7 | 8 | 3 | 103,53 | 102,817 |  |
| 378 | 462,524 | 2,7865 | -2.237 | 33 | 59 | 10 | 10 | 5 | 126,59 | 129,38 |  |
| 379 | 444,547 | 3,0349 | -4.793 | 27 | 42 | 9 | 12 | 3 | 195,23 | 108,875 |  |
| 380 | 322,361 | 2,519 | -3.421 | 24 | 44 | 7 | 6 | 3 | 87,74 | 91,8414 |  |
| 381 | 331,389 | 2,5295 | -3.492 | 23 | 42 | 6.75 | 7 | 3 | 102,02 | 92,5122 |  |
| 382 | 217,178 | 1,0693 | -0.565 | 16 | 25 | 5 | 5 | 3 | 68,39 | 56,0192 |  |
| 383 | 395,433 | 0,0822 | -3.639 | 27 | 50 | 8.75 | 11 | 3 | 115,12 | 105,841 |  |
| 384 | 335,378 | 2,5154 | -3.137 | 23 | 42 | 7.5 | 8 | 3 | 111,78 | 84,5547 |  |
| 385 | 337,351 | 1,0631 | -2.919 | 23 | 39 | 7.2 | 9 | 2 | 127,76 | 83,8287 |  |
| 386 | 335,421 | 2,365 | -2.623 | 23 | 46 | 8 | 7 | 3 | 71 | 90,0715 |  |
| 387 | 334,37 | 2,1499 | -3.363 | 24 | 47 | 7.5 | 8 | 2 | 114,33 | 92,4056 |  |
| 388 | 332,417 | 3,5266 | -3.621 | 23 | 44 | 7 | 6 | 2 | 74,86 | 91,9032 |  |
| 389 | 306,704 | 1,7019 | -3.703 | 21 | 33 | 7.5 | 8 | 2 | 94,07 | 74,9162 |  |
| 390 | 295,357 | 3,0373 | -2.785 | 20 | 38 | 6.5 | 7 | 2 | 106,46 | 75,7237 |  |
| 391 | 274,341 | 2,5363 | -4.35 | 19 | 34 | 5.25 | 6 | 2 | 98,14 | 77,7476 |  |
| 392 | 325,405 | 2,51728 | -4.914 | 24 | 50 | 5.5 | 5 | 4 | 75,75 | 90,881 |  |
| 393 | 232,235 | 0,80328 | -1.654 | 17 | 29 | 6.5 | 5 | 1 | 70,4 | 59,9535 |  |
| 394 | 329,307 | 2,167 | -3.273 | 24 | 41 | 8 | 8 | 3 | 106,45 | 86,7939 |  |
| 395 | 321,842 | 4,319 | -2.588 | 22 | 46 | 3.45 | 4 | 2 | 41,49 | 92,5815 |  |
| 396 | 281,753 | 4,317 | -2.97 | 19 | 36 | 2.25 | 4 | 2 | 21,26 | 76,9187 |  |
| 397 | 327,383 | 0,7909 | -3.643 | 21 | 35 | 9 | 10 | 2 | 152,54 | 78,3757 |  |
| 398 | 403,426 | 2,2518 | -4.399 | 29 | 54 | 7.25 | 9 | 3 | 78,43 | 110,153 |  |
| 399 | 464,464 | 3,1581 | -2.993 | 32 | 53 | 10 | 12 | 3 | 118,31 | 112,589 |  |
| 400 | 236,224 | 1,7624 | -1.356 | 17 | 30 | 5.75 | 6 | 2 | 66,61 | 59,21 |  |
| 401 | 335,363 | 1,0226 | -3.5 | 25 | 45 | 8.5 | 8 | 4 | 80,04 | 98,1175 |  |
| 402 | 370,49 | 2,3088 | -1.456 | 24 | 49 | 10 | 9 | 4 | 118,84 | 91,759 |  |
| 403 | 376,435 | 4,0173 | -5.493 | 27 | 47 | 8 | 8 | 5 | 107,82 | 101,515 |  |
| 404 | 320,364 | 1,4687 | -3.227 | 22 | 39 | 7 | 7 | 2 | 105,64 | 82,8087 |  |
| 405 | 371,472 | 2,9834 | -1.381 | 24 | 46 | 9.5 | 8 | 2 | 125,43 | 93,5243 |  |
| 406 | 386,508 | 4,0352 | -3.986 | 27 | 55 | 8.5 | 6 | 3 | 74,86 | 111,394 |  |
| 407 | 387,499 | 2,7864 | -5.496 | 27 | 55 | 8.2 | 8 | 4 | 97,58 | 109,839 |  |
| 408 | 413,493 | 2,6876 | -3.241 | 29 | 54 | 8.5 | 9 | 3 | 122,92 | 111,59 |  |
| 409 | 285,298 | 1,9619 | -3.813 | 21 | 37 | 7 | 6 | 2 | 81,18 | 77,0772 |  |
| 410 | 289,33 | 2,385 | -3.718 | 21 | 41 | 5.5 | 6 | 2 | 87,24 | 80,6414 |  |
| 411 | 304,172 | 2,3899 | -3.495 | 19 | 35 | 5 | 7 | 2 | 63,99 | 74,3157 |  |
| 412 | 331,393 | 1,8756 | -4.516 | 23 | 42 | 5.5 | 8 | 3 | 105,42 | 94,6787 |  |
| 413 | 343,377 | 3,1994 | -4.776 | 25 | 47 | 6.75 | 7 | 2 | 100,29 | 95,9381 |  |
| 414 | 415,443 | 0,9384 | -4.1 | 30 | 57 | 9 | 10 | 3 | 109,38 | 111,74 |  |
| 415 | 276,291 | 0,1727 | -4.582 | 20 | 37 | 3.5 | 7 | 2 | 78,15 | 75,5657 |  |
| 416 | 319,36 | 2,8505 | -4.154 | 24 | 44 | 4.5 | 6 | 4 | 75,08 | 91,7194 |  |
| 417 | 301,32 | 3,1763 | -3.804 | 21 | 34 | 6.75 | 7 | 3 | 119,48 | 76,6849 |  |
| 418 | 328,185 | 3,1653 | -2.263 | 18 | 29 | 6 | 7 | 2 | 107,31 | 71,6764 |  |
| 419 | 405,476 | 2,3015 | -3.912 | 29 | 52 | 8.5 | 9 | 5 | 108,28 | 118,481 |  |
| 420 | 412,482 | 3,2891 | -5.133 | 30 | 61 | 4.5 | 8 | 5 | 117,34 | 110,933 |  |
| 421 | 377,436 | 3,3553 | -4.607 | 28 | 54 | 8 | 6 | 4 | 78,51 | 110,243 |  |
| 422 | 353,415 | 2,3715 | -4.452 | 26 | 52 | 6.5 | 6 | 4 | 69,72 | 109,241 |  |
| 423 | 360,477 | 3,4601 | -5.448 | 25 | 52 | 7 | 8 | 4 | 102,93 | 98,2727 |  |
| 424 | 379,431 | 0,8091 | -3.792 | 26 | 49 | 9.7 | 9 | 3 | 114,76 | 105,858 |  |
| 425 | 425,481 | 2,039 | -3.836 | 31 | 61 | 10 | 9 | 4 | 115,47 | 123,043 |  |
| 426 | 384,447 | 2,7091 | -4.227 | 26 | 51 | 9.75 | 9 | 2 | 110,39 | 99,9717 |  |
| 427 | 314,357 | 1,7662 | -2.374 | 21 | 40 | 9.45 | 8 | 2 | 93,32 | 80,5437 |  |
| 428 | 351,401 | 1,3394 | -3.123 | 23 | 38 | 8.5 | 9 | 3 | 139,15 | 88,056 |  |
| 429 | 304,367 | 2,2116 | -3.828 | 21 | 38 | 7.5 | 7 | 2 | 102,18 | 81,8402 |  |
| 430 | 412,509 | 2,9115 | -4.487 | 29 | 56 | 8.75 | 9 | 4 | 112,04 | 113,927 |  |
| 431 | 365,47 | 3,3103 | -3.518 | 24 | 45 | 8 | 8 | 3 | 115,6 | 93,9235 |  |
| 432 | 253,298 | 0,9127 | -2.128 | 18 | 37 | 6 | 6 | 1 | 78,51 | 74,5284 |  |
| 433 | 278,261 | 0,8884 | -2.333 | 20 | 35 | 7.5 | 7 | 2 | 90,65 | 71,6348 |  |
| 434 | 278,261 | 0,8884 | -2.333 | 20 | 35 | 7.5 | 7 | 2 | 90,65 | 71,6348 |  |
| 435 | 411,701 | 5,1107 | -5.284 | 23 | 36 | 4.5 | 7 | 3 | 91,32 | 97,3748 |  |
| 436 | 435,472 | 2,4982 | -5.189 | 32 | 61 | 9.5 | 8 | 5 | 91,14 | 126,703 |  |
| 437 | 351,155 | 0,9775 | -3.061 | 21 | 34 | 6.5 | 8 | 3 | 84,3 | 86,3957 |  |
| 438 | 298,74 | 1,9186 | -2.629 | 20 | 37 | 6 | 6 | 2 | 40,62 | 82,6575 |  |
| 439 | 298,74 | 1,9186 | -2.629 | 20 | 37 | 6 | 6 | 2 | 40,62 | 82,6575 |  |
| 440 | 478,177 | 0,1365 | -5.84 | 26 | 46 | 3.2 | 6 | 4 | 46,11 | 108,935 |  |
| 441 | 339,453 | 2,7314 | -1.919 | 23 | 49 | 8 | 7 | 2 | 78,1 | 92,4007 |  |
| 442 | 376,409 | 1,4075 | -4.434 | 28 | 51 | 8 | 7 | 4 | 75,51 | 113,104 |  |
| 443 | 310,438 | 2,4154 | -3.457 | 20 | 39 | 5.5 | 7 | 2 | 113,35 | 82,5647 |  |
| 444 | 269,34 | 1,5268 | -2.566 | 19 | 42 | 6 | 6 | 1 | 78,51 | 79,8094 |  |
| 445 | 325,345 | 3,2026 | -3.196 | 23 | 37 | 7 | 8 | 4 | 108,07 | 83,125 |  |
| 446 | 377,415 | 2,632 | -2.588 | 26 | 48 | 9.2 | 9 | 4 | 121,01 | 93,1397 |  |
| 447 | 225,311 | 2,068 | -1.641 | 15 | 30 | 4.5 | 5 | 1 | 94,17 | 61,1724 |  |
| 448 | 390,316 | 2,1241 | -4.783 | 28 | 43 | 6.5 | 10 | 3 | 95,16 | 93,9689 |  |
| 449 | 310,415 | 2,1435 | -1.538 | 21 | 45 | 8 | 7 | 3 | 96,56 | 84,6397 |  |
| 450 | 216,163 | 1,9071 | -1.181 | 15 | 23 | 4 | 7 | 2 | 56,21 | 45,6624 |  |
| 451 | 326,414 | 2,8474 | -5.25 | 22 | 45 | 6 | 8 | 2 | 111,41 | 83,7957 |  |
| 452 | 290,364 | 2,1467 | -3.079 | 19 | 31 | 6 | 7 | 3 | 113,69 | 76,103 |  |
| 453 | 332,381 | 0,4061 | -3.619 | 23 | 41 | 7 | 9 | 3 | 112,9 | 88,485 |  |
| 454 | 391,485 | 3,1153 | -4.225 | 27 | 54 | 8 | 8 | 3 | 123,82 | 110,032 |  |
| 455 | 266,276 | 1,352 | -2.401 | 18 | 29 | 7.75 | 8 | 2 | 125,36 | 63,8119 |  |
| 456 | 357,404 | 1,7696 | -4.525 | 26 | 51 | 7 | 7 | 3 | 81,5 | 101,897 |  |
| 457 | 438,503 | 1,3875 | -3.928 | 31 | 57 | 9.5 | 10 | 5 | 123,26 | 123,443 |  |
| 458 | 443,948 | 4,4718 | -3.805 | 27 | 44 | 10 | 11 | 4 | 164,08 | 105,735 |  |
| 459 | 435,545 | 2,4376 | -4.536 | 31 | 60 | 8.5 | 9 | 5 | 105,34 | 126,336 |  |
| 460 | 407,419 | 1,9937 | -4.424 | 30 | 55 | 7.5 | 8 | 5 | 96,97 | 113,387 |  |
| 461 | 359,463 | 1,9163 | -1.946 | 26 | 57 | 8.2 | 6 | 3 | 61,88 | 107,998 |  |
| 462 | 416,476 | 2,4461 | -4.426 | 31 | 59 | 8 | 8 | 5 | 81,67 | 131,599 |  |
| 463 | 236,267 | 1,7801 | -1.605 | 17 | 33 | 5.75 | 5 | 1 | 81,42 | 63,2586 |  |
| 464 | 357,448 | 1,8828 | -2.103 | 23 | 44 | 9.7 | 9 | 3 | 122,28 | 89,3707 |  |
| 465 | 372,441 | 2,9386 | -5.879 | 26 | 49 | 6.75 | 8 | 4 | 111,41 | 96,9872 |  |
| 466 | 296,779 | 3,3011 | -3.488 | 19 | 33 | 5 | 8 | 3 | 113,65 | 78,1191 |  |
| 467 | 467,412 | 1,4412 | -1.411 | 32 | 57 | 7.25 | 13 | 2 | 119,41 | 111,093 |  |
| 468 | 427,921 | 3,9339 | -3.3 | 28 | 53 | 8.5 | 9 | 3 | 80,65 | 106,63 |  |
| 469 | 359,443 | 3,0896 | -3.483 | 25 | 49 | 8.5 | 7 | 4 | 80,65 | 95,1985 |  |
| 470 | 283,3 | 2,7429 | -2.982 | 21 | 37 | 5 | 5 | 3 | 57,78 | 79,3834 |  |
| 471 | 479,434 | 1,2865 | -3.252 | 34 | 60 | 8.2 | 12 | 3 | 170,08 | 114,912 |  |
| 472 | 285,341 | 2,3054 | -3.098 | 21 | 42 | 6 | 5 | 3 | 74,85 | 81,8664 |  |
| 473 | 334,417 | 3,1541 | -4.633 | 22 | 39 | 7 | 8 | 4 | 138,21 | 86,7027 |  |
| 474 | 448,45 | 3,2553 | -4.776 | 31 | 53 | 8.75 | 12 | 3 | 163,25 | 108,497 |  |
| 475 | 360,474 | 2,9733 | -4.826 | 25 | 51 | 7 | 7 | 3 | 95,47 | 104,786 |  |
| 476 | 376,369 | 1,6588 | -3.83 | 28 | 48 | 8.75 | 9 | 5 | 102,24 | 104,299 |  |
| 477 | 289,31 | 1,7583 | -1.99 | 20 | 33 | 7 | 7 | 3 | 105,37 | 74,5107 |  |
| 478 | 452,549 | 3,5902 | -4.829 | 31 | 54 | 7.5 | 9 | 4 | 154,77 | 122,412 |  |
| 479 | 333,344 | 2,6341 | -3.193 | 25 | 43 | 7.5 | 7 | 4 | 85,32 | 90,5527 |  |
| 480 | 381,382 | 2,388 | -4.581 | 28 | 50 | 9 | 8 | 4 | 99,93 | 109,545 |  |
| 481 | 299,71 | 1,0041 | -3.98 | 20 | 34 | 6.75 | 8 | 1 | 96,53 | 72,6811 |  |
| 482 | 258,273 | 0,9384 | -2.236 | 19 | 35 | 7.2 | 5 | 3 | 62,4 | 74,9662 |  |
| 483 | 228,247 | 1,8112 | -2.881 | 17 | 31 | 5 | 4 | 3 | 61,96 | 65,0649 |  |
| 484 | 298,3 | 0,9112 | -3.11 | 22 | 39 | 7 | 8 | 4 | 91,15 | 85,1182 |  |
| 485 | 386,531 | 2,1836 | -1.659 | 28 | 64 | 9.5 | 6 | 3 | 55,89 | 124,776 |  |
| 486 | 449,459 | 2,503 | -5.826 | 33 | 59 | 9.25 | 10 | 4 | 122,63 | 127,52 |  |
| 487 | 444,478 | 2,3526 | -4.448 | 32 | 61 | 5.75 | 9 | 3 | 116,61 | 125,039 |  |
| 488 | 264,3 | 2,1691 | -2.451 | 18 | 31 | 4.75 | 6 | 2 | 99,58 | 66,6839 |  |
| 489 | 358,435 | 1,7648 | -3.645 | 26 | 55 | 10 | 7 | 4 | 65,12 | 112,97 |  |
| 490 | 490,594 | 3,3905 | -5.01 | 36 | 74 | 9.5 | 8 | 5 | 90,03 | 152,513 |  |
| 491 | 325,362 | 1,5671 | -3.15 | 24 | 45 | 6 | 6 | 3 | 78,51 | 95,5794 |  |
| 492 | 264,109 | 1,4672 | -2.382 | 16 | 27 | 5 | 7 | 1 | 63,99 | 62,0087 |  |
| 493 | 266,319 | 1,4836 | -2.261 | 18 | 34 | 6.7 | 7 | 3 | 91,31 | 71,1537 |  |
| 494 | 266,319 | 1,4836 | -2.261 | 18 | 34 | 6.7 | 7 | 3 | 91,31 | 71,1537 |  |
| 495 | 277,362 | 2,1591 | -3.19 | 20 | 44 | 6 | 5 | 2 | 74,85 | 79,0994 |  |
| 496 | 394,404 | 3,9244 | -4.403 | 28 | 46 | 8.5 | 9 | 5 | 121,6 | 99,155 |  |
| 497 | 291,302 | 1,7712 | -3.461 | 21 | 39 | 6.75 | 7 | 2 | 74,61 | 77,1792 |  |
| 498 | 360,451 | 2,7541 | -2.714 | 26 | 55 | 6.25 | 7 | 2 | 90,54 | 104,721 |  |
| 499 | 364,395 | 2,338 | -4.237 | 25 | 43 | 6.5 | 9 | 2 | 128,43 | 90,3211 |  |
| 500 | 414,498 | 2,2582 | -5.139 | 30 | 63 | 8.25 | 8 | 4 | 82,19 | 127,591 |  |

Figure S3 **Distribution of molecular properties of the 500 fragments.** Molecular weight (MW), LogP, LogS, number of heavy atoms (HAC), hydrogen bonds donors (HD), hydrogen bonds acceptors (HA), number of hetero atoms (nHA), number of rings (NR), polar surface area (PSA) and molar refractivity (MR).
