# Supplementary material for: Virtual and Biophysical Screening Targeting the γ-Tubulin Complex – A New Target for the Inhibition of Microtubule Nucleation
Source: PLoS One. 2013 May 15;8(5):e63908. doi: 10.1371/journal.pone.0063908 (PMC3655011; doi:10.1371/journal.pone.0063908)
Supplement: Figure S4 — NMR spectra of the best four hits. The effects of the best hits from docking on the melting temperature Tm of the complex are shown in Fig. 8 of the paper. This figure presents the NMR spectra for a subset of the ligands that had the most pronounced effect on the complex, i.e. whose Tm varied by at least 2°C. a. NM372. b. NM156. c. NM87. d. NM442. The 1D spectra have been acquired on a Bruker spectrometer operating at the proton frequency of 600 MHz, using a cryoprobe. Asterisks denote the solvent peaks: DMSO at 2.50 ppm and the residual HDO signal at 3.30 ppm. Molecules shown in insets have been drawn with Marvin 5.11.3, 2012, ChemAxon (http://www.chemaxon.com). (DOCX) [file pone.0063908.s004.docx]

Figure S4 **NMR spectra of the best four hits**. The effects of the best hits from docking on the melting temperature T_m_ of the complex are shown in Fig. 8 of the paper. This figure presents the NMR spectra for a subset of the ligands that had the most pronounced effect on the complex, i.e. whose T_m_ varied by at least 2°C. a. NM372. b. NM156. c. NM87. d. NM442. The 1D spectra have been acquired on a Bruker spectrometer operating at the proton frequency of 600 MHz, using a cryoprobe. Asterisks denote the solvent peaks: DMSO at 2.50 ppm and the residual HDO signal at 3.30 ppm. Molecules shown in insets have been drawn with Marvin 5.11.3, 2012, ChemAxon (http://www.chemaxon.com).
